# Self‐Powered Autonomous Electrostatic Dust Removal for Solar Panels by an Electret Generator

**DOI:** 10.1002/advs.202401689

**Published:** 2024-05-05

**Authors:** Rong Ding, Zeyuan Cao, Junchi Teng, Yujia Cao, Xiaoyu Qian, Wei Yue, Xiangzhu Yuan, Kang Deng, Zibo Wu, Shuiqing Li, Liwei Lin, Xiongying Ye

**Affiliations:** ^1^ State Key Laboratory of Precision Measurement Technology and Instruments Department of Precision Instrument Tsinghua University Beijing 100084 China; ^2^ Key Laboratory for Thermal Science and Power Engineering of Ministry of Education Department of Energy and Power Engineering Tsinghua University Beijing 100084 China; ^3^ Berkeley Sensor and Actuator Center and Department of Mechanical Engineering University of California at Berkeley Berkeley CA 94720 USA

**Keywords:** dust removal, electret generator, electrostatic, solar panel, water adsorption

## Abstract

Solar panels often suffer from dust accumulation, significantly reducing their output, especially in desert regions where many of the world's largest solar plants are located. Here, an autonomous dust removal system for solar panels, powered by a wind‐driven rotary electret generator is proposed. The generator applies a high voltage between one solar panel's output electrode and an upper mesh electrode to generate a strong electrostatic field. It is discovered that dust particles on the insulative glass cover of the panel can be charged under the high electrical field, assisted by adsorbed water, even in low‐humidity environments. The charged particles are subsequently repelled from the solar panel with the significant Coulomb force. Two panels covered with sand dust are cleaned in only 6.6 min by a 15 cm diameter rotary electret generator at 1.6 m s^−1^ wind speed. Experimental results manifest that the system can work effectively in a wide range of environmental conditions, and doesn't impact the panel performance for long‐term operation. This autonomous system, with its high dust removal efficiency, simplicity, and low cost, holds great potential in practical applications.

## Graphical abstract

An autonomous dust removal system for solar panels, powered by a wind‐driven rotary electret generator is proposed. The system has the advantages of low cost, simple structure, and excellent dust removal performances, while it has minimum interferences to the panel performance. With significant potential for addressing dust accumulation problems on solar panels in desert regions, it offers a promising solution.

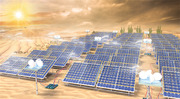

## 1 Introduction

Climate change is a critical and urgent threat to human beings and the planet.^[^ ^1^, ^2^, ^3^ ^]^ On 13 December 2023 at the COP28 UN Climate Conference, the United Arab Emirates Consensus was delivered to include an unprecedented reference for transitioning away from all fossil fuels.^[^ ^4^ ^]^ Therefore, carbon‐free renewable energy is very significant for net zero carbon emissions by 2050.^[^ ^5^, ^6^, ^7^ ^]^ According to the outlook delivered by the International Energy Agency,^[^ ^8^, ^9^ ^]^ the share of renewables such as solar, wind, and water energy in electricity generation will rise from 28% in 2021 to ≈50% in 2030 and 80% by 2050, and the solar energy capacity will expand from 151 gigawatts (GW) in 2021 to 370 GW in 2030 and almost 600 GW in 2050 to have the largest share.

Tremendous efforts have been made in the field of solar energy technology to improve the conversion efficiency gradually approaching its intrinsic limit.^[^ ^10^ ^]^ However, the accumulation of dust on solar panels can block sunlight and reduce the output power, which is a significant obstacle to further improving solar energy conversion efficiency.^[^ ^11^, ^12^ ^]^ In particular, to ensure sufficient land and sunlight, many of the world's largest solar plants are located in sparsely populated desert regions where the accumulation of dust can be significant, such as Junma Solar Power Station in Kubuqi desert (China), Tengger Desert Solar Park (China), Solar Energy Generating Systems in Mojave Desert (United States), Bhadla Solar Park (India), Benban Solar Park (Egypt), and Al Kharsaah Solar Power Plant (Qatar).^[^ ^13^ ^]^ According to the literature,^[^ ^14^ ^]^ when dust deposition density increased from 0 to 22 g m^−2^, the corresponding reduction of output efficiency grew from 0 to 26%. For example, a photovoltaic module in Doha will only be able to provide ≈85% of the electricity if it is not cleaned for one month.^[^ ^15^ ^]^ At present, dust removal relies on manual cleaning with water and simple tools, resulting in low operational efficiency, fresh water consumption, high labor costs, and potential risks to the safety of cleaning workers.^[^ ^16^ ^]^ Several prior efforts have been carried out for dust removals, such as robotic cleaning,^[^ ^17^, ^18^ ^]^ anti‐dust surface coating,^[^ ^19^, ^20^, ^21^ ^]^ and electrostatic cleaning,^[^ ^22^, ^23^ ^]^ etc. Several issues arise with the schemes of robotic mechanical cleanings and anti‐dust surface coatings, such as high equipment cost, contact scrubbing damage, limited durability, and reliability.

Electrodynamic screen (EDS) is a popular electrostatic dust removal system proposed by F.B. Tatom and collaborators at NASA in 1967^[^ ^24^ ^]^ and further developed by Masuda at the University of Tokyo in the 1970s.^[^ ^25^, ^26^ ^]^ Specifically, the EDS system has been successfully implemented in solar panels on Mars rovers.^[^ ^23^ ^]^ It generally consists of a mesh electrode embedded in the glass cover of the solar panel, and a high‐voltage power supply system to generate high‐voltage AC traveling or standing waves to remove dust by the dielectrophoretic (DEP) force.^[^ ^27^ ^]^ An improved detachable EDS system was proposed,^[^ ^28^ ^]^ based on the natural charging of dust particles and Coulomb force. In 2022, Panat, S. et. al proposed a moisture–assisted charging approach based on the Coulombic force to repel charged dust particles on the solar panel,^[^ ^29^ ^]^ with a high DC voltage between an upper moving plate electrode and a lower transparent electrode to charge the moisture‐adsorbed particles and remove them. These systems generally require a control system to regulate the mechanical movements resulting in high complexity, cost, and possible mechanical failure. Therefore, reducing the complexity, cost, and energy consumption of the electrostatic cleaning system are major challenges.

Recently, electrostatic generators including triboelectric generators and electret generators,^[^ ^30^, ^31^, ^32^, ^33^, ^34^ ^]^ have been proven to be able to harvest energy from low‐frequency mechanical motions effectively. Electrostatic generators can easily generate a high‐voltage output in thousands of volts as a unique superiority in high‐voltage devices.^[^ ^35^, ^36^, ^37^, ^38^, ^39^ ^]^ Moreover, rotary electret generators (REGs) can operate at very low wind speeds,^[^ ^40^ ^]^ as a good potential candidate in dust removal systems for solar panels by harvesting the wind energy without extra power supplies.

**Figure 1:**
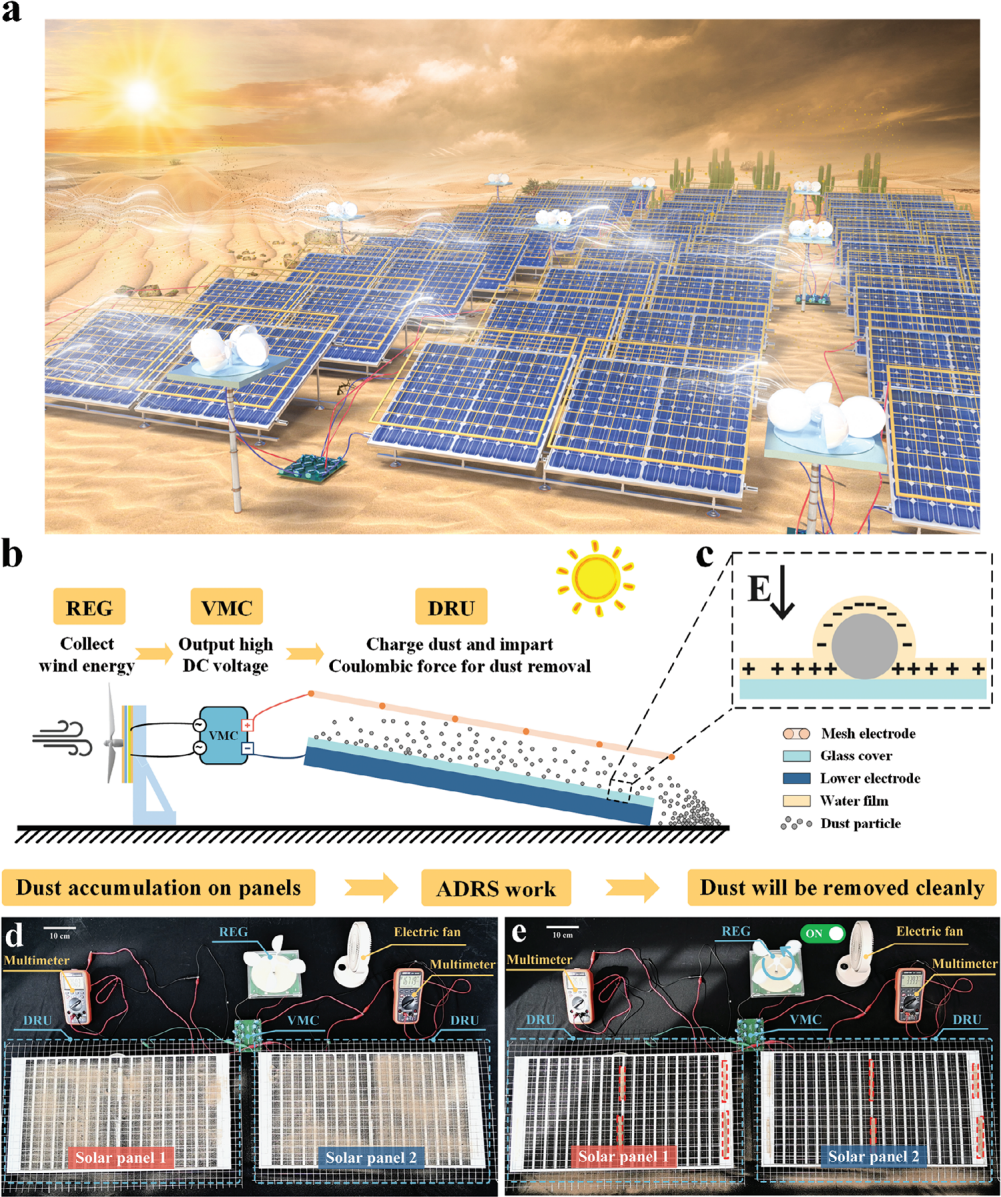
Overview of ADRS. a) Application scenario of the ADRS. b) Schematics of the ADRS. c) Schematic of the charged particle on the glass cover due to the adsorbed water films. Comparison of the solar panel surface: d) before, and e) after the dust removal process driven by the wind‐powered energy generator. The red frames in Figure 1e represent the dust particles that have settled on the support structure.

In this work, a self‐powered autonomous dust removal system (ADRS) for solar panels is proposed as shown in **Figure** **1**a. The ADRS consists of a wind‐driven REG, a voltage multiplying circuit (VMC) to generate higher DC voltage from the AC outputs of REG, and a dust removal unit (DRU) that consists of an upper mesh electrode mounted on the solar panel and one output electrode of the solar panel under the glass cover of the panel. A primary component in dust particles and glass cover is silica, which is particularly prone to adsorb moisture to form water films even in low‐humidity environments. As such, the dust particles on the glass cover can be easily charged by the separation of ions within the water film under an electric field and repelled via strong Coulombic force. We investigated the charging mechanism of the dust particles and the impact factors associated with the water film, the carried charge and the repelling force on dust particles. The prototype ADRS is optimized and results show two DRUs can thoroughly remove massive sand dust on two solar panels of 67 × 35 cm in just 6.6 min driven by one REG with a diameter of 15 cm under a low wind speed of 1.6 m s^−1^. After a 70 day long‐term operational test, the prototype ADRS solar panel showed consistent performances to have enormous potential for practical applications.

## 2 Results

### 2.1 Overview of ADRS

The schematic of the structure of the ADRS is shown in Figure 1b, comprising a REG to convert wind energy to electric energy with high AC voltage outputs, a VMC to transfer the AC voltage to a higher DC voltage, and a DRU to complete the dust removal process. The DRU is composed of an upper mesh electrode and a lower electrode that is the output electrode of the solar panel covered by a glass plate. The primary component of the dust particles and the glass cover is silica that is particularly prone to absorb moisture to form a water film on the surface. Due to the electric field in the DRU, charge transfer occurs between the water films of the dust particles and the glass cover to electrically charge the dust particles, as shown in Figure 1c. The Coulombic force is generated in the DRU to repel charged dust particles from the solar panel surface as they slide from the tilted panel to the ground due to the gravity force. Figure 1d,e shows the comparison of the solar panel surface before and after the operation of the ADRS. It can be observed that most dust on the solar panels is removed.

This article is divided into the following parts. First, the charging mechanism of the dust particles on a glass cover under an electric field is studied and analyzed. Second, factors associated with the water film on dust removal are investigated, including relative humidity, cover surface materials, and particle types. Third, the output characteristics of the REG and voltage on the DRU are characterized. Fourth, the parameters affecting DRU, dust particle size, and desert dust types and their removal performance are discussed. Finally, a demonstration test with the dust removal effect with respect to the impact of the high DC voltage on solar panel output is presented.

### 2.2 Analysis of Particle Charging Mechanism and Influencing Factors

**Figure 2:**
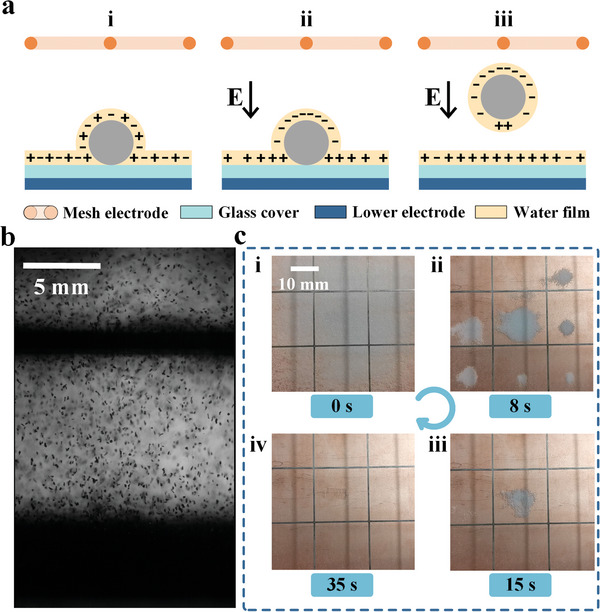
Schematic of the dust charging mechanism and verification. a) Charging process of a particle on a glass cover under an electric field. Here, the particle diameter and the thickness of the water film are drawn with exaggerated sizes. b) Photograph of the repelled particles from the glass cover. c) Sequential photographs of dust on the glass cover mounted with a mesh electrode during the dust removal process.

The primary component of desert dust particles and the glass cover of solar panels are made of silica, which is particularly prone to adsorb moisture and form a water film, even in a low humidity environment.^[^ ^41^, ^42^, ^43^ ^]^ There are dissolved positive and negative ions naturally in the water film (**Figure** **2**a‐i), where the negative and positive ions may move and accumulate on the particle and glass cover,^[^ ^44^, ^45^ ^]^ respectively, under a downward electric field to charge the particle negatively (Figure 2a‐ii). When the accumulated charge on the particle reaches a threshold as the overall upward force is greater than the downward force, the particle can bounce off the glass‐cover surface (Figure 2a‐iii). Conversely, if an upward electric field is applied, the particle accumulates positive charges and can also be repelled upward as shown in Figure S1 (Supporting Information).

The aforementioned charging mechanism of dust particles is experimentally tested by using silica particles of ≈110 µm in effective diameter in an environment with a relative humidity of 30%. Figure 2b and Movie S1 (Supporting Information) show a photograph and video of particles bouncing off the glass cover when a high voltage of 4.5 kV from a power supply is applied between the upper mesh electrode and the electrode under the glass cover. The schematic and details of the observation setup are illustrated in Figure S2 (Supporting Information). For other experiments, the VMC converts the output of the REG into high DC voltage applied to the DRU to realize particles bouncing, as shown in Figure 1b.

Figure 2c shows the gradual removal process of particles on the glass cover with the ADRS, and the total process is shown in Movie S2 (Supporting Information). Here, the DRU is simplified to consist of an upper mesh electrode and a lower plate electrode covered with a thin glass plate, and the DRU is tilted with an angle of 10 degrees and the mesh pitch is 20 mm. After the REG starts, the particles are gradually removed from the glass cover under the mesh electrode from the central region to the edge. This indicates that after applying high voltage for a certain time, particles on the glass cover have been highly charged, and the stronger the electric field, the faster the dust charging and removal process.

**Figure 3:**
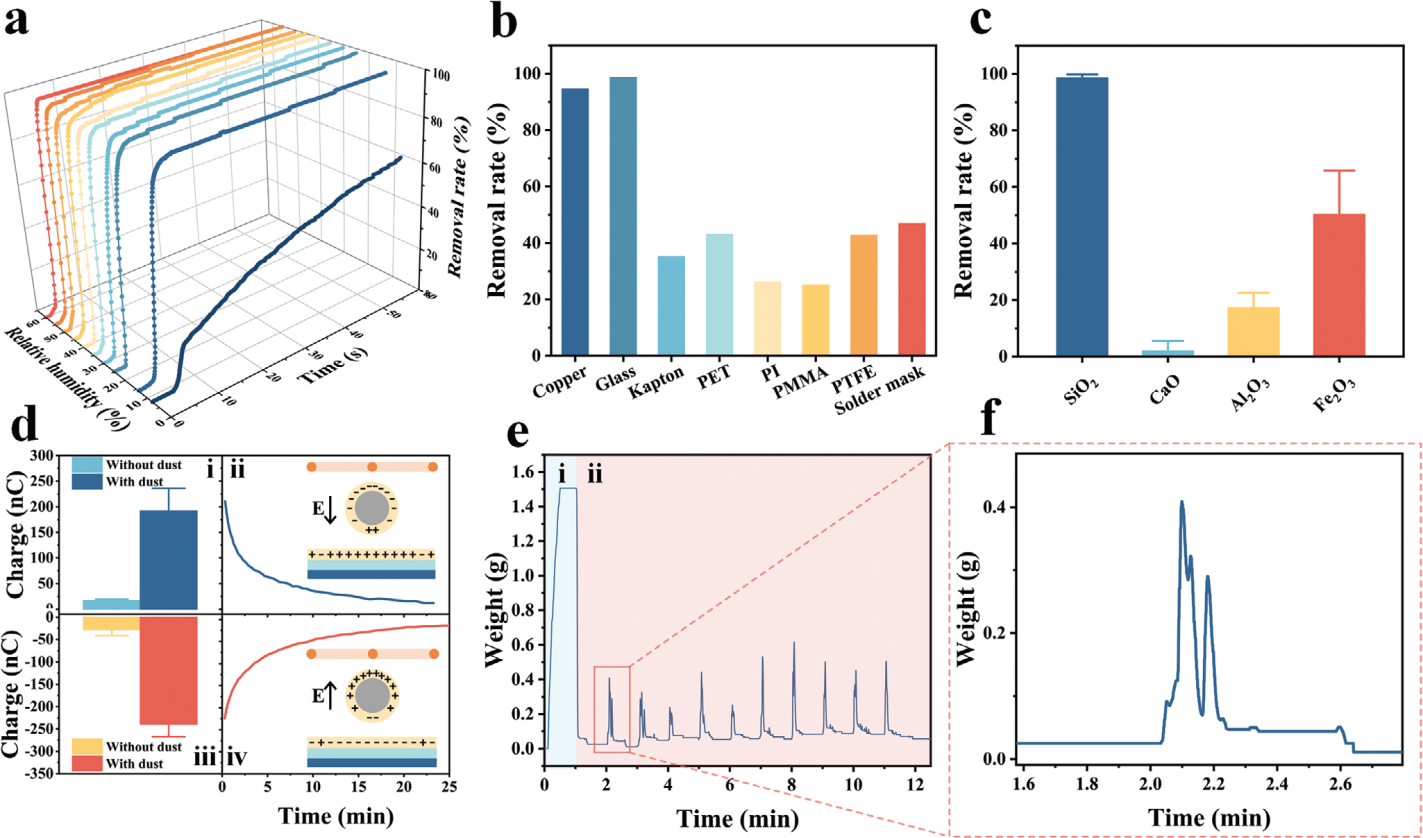
Experimental results for further verification of the dust charging process based on the absorbed water film. Dust removal rate under a) different relative humidity, b) different materials covering the lower electrode and c) different particle materials. d) Surface charge on the glass cover. i/iii Delta charge before and after the ADRS operation; ii/iv Charge variation versus time after the dust removal. e) Result of continuous dust removal tests, and f) an enlarged view during the dust spreading process.

To investigate the factors associated with the particle charging, namely, the water film on dust removal, we tested the dust removal efficiency with the ADRS under different conditions with results shown in **Figure** **3**a–c. Figures 3a and S3a (Supporting Information) show the dust removal rate and the removal time by using the Ulanbuhe‐1 desert sand under different relative humidities. The removal rate is defined as:

(1) $$\mathrm{Removal}\;\mathrm{rate}=\frac{\mathrm{initial}\;\mathrm{mass}-\mathrm{remained}\;\mathrm{mass}}{\mathrm{initial}\;\mathrm{mass}}\times 100\%$$

The removal rate at 60 s and the time reaching the half removal rate (*T_half_*) are used to evaluate the dust removal effect. This experiment is conducted in a fume cupboard with a humidifier. It can be found that the dust removal effect has little change as the humidity increases from 14% to 60%. However, when the relative humidity decreases to 8%, the dust removal speed slows down noticeably, and the dust removal rate at 60 s is only ≈60%, significantly lower than those in higher humidity environments. As the dust removal time extends to 300 s, the dust removal rate gradually reaches over 90%, as depicted in Figure S3b (Supporting Information). This is because the water film persists, even at very low humidity, in the form of a very thin and/or discontinuous layer,^[^ ^41^, ^45^ ^]^ which hinders the removal process. It is worth noting that excessively high humidity could lead to problems such as electrical shorting or air breakdown, thereby reducing the efficiency of the dust removal system.

The dust removal rates for silica particles on different materials covering the lower electrode are shown in Figures 3b and S3c (Supporting Information) with copper as the case of no additional covering material. The dust removal rates at 60 s exceed 94% for copper and glass. For materials like Kapton, PET, PI, PMMA, PTFE, and Solder mask, the dust removal rates at 60 s are below 50%. Copper is a conductor such that charges can be directly transferred from copper to the water film on the particles^[^ ^29^ ^]^ to result in a high dust removal rate. For insulating materials, glass has significantly better hydrophilicity as compared to those of other materials to adsorb more moisture for a thicker water film to facilitate the dust charging process. Figures 3c and S4 (Supporting Information) show the dust removal rates of particles ≈110 µm in diameter made of different materials on the glass cover. It can be found that SiO_2_ exhibits a significantly higher removal rate when compared to those of particles made of CaO, Al_2_O_3_, and Fe_2_O_3_. This is attributed to the high hydrophilicity of SiO_2_ to make it easier to form water films and acquire charges. In addition to the adsorbed water film, various properties of the cover and particle material such as surface free energy, surface roughness, relative permittivity, etc. all affect the adhesion forces acting on the particles, resulting in the dust removal efficiency not being linearly related to the hydrophilicity.

In order to characterize the charge‐moving process, the surface potentials of the glass cover with and without dust are measured before and after the ADRS operation, and the surface charges are calculated based on the surface potentials, as shown in Figure 3d and Table S1 (Supporting Information), with the details of the experiments and surface charge calculation shown in Note S1 (Supporting Information). It is observed that with dust, the change of the surface charge on the glass before and after ADRS operation is significant. Without dust, there are no noticeable surface charge variations before and after the ADRS operation (Figure 3d‐i,iii). Furthermore, the polarity of the increased charge matches the polarity of the voltage connected to the upper mesh electrode, which is consistent with the aforementioned analysis. When the ADRS stops, the charge on the glass surface gradually decreases as shown in Figure 3d‐ii,iv, indicating that the charge remaining on the surface of the glass cover can be dissipated quickly. The mass changes of the DRU during the continuous operation of ADRS are measured as shown in Figure 3e. At stage i, the ADRS is turned off and ≈1.5 g of dust is evenly spread on the glass surface. At stage ii, the ADRS is turned on continuously to spread ≈1.5 g of dust on the glass surface approximately every minute. It can be seen that dust is promptly removed, as illustrated in Figure 3f. After 10 min of continuous operation, the ADRS still effectively removes dust, indicating that despite some charges accumulated in the water film of the glass cover, the dust removal process can continue. This might be attributed to the rapid dispersion of charges in the water film on the glass, particularly at the edges to prevent the saturation of charges for the sustained dust removal process.

The forces to repel the dust particles resting on the horizontal glass surface are evaluated. The vertical z‐directional net force (*F_N_*) acting on a particle can be presented as the following equation:

(2) $$F_{N}=\left(\vec{F_{E}}+\vec{F_{D}}+\vec{F_{I}}+\vec{F_{V}}+\vec{F_{C}}+\vec{F_{G}}\right)\cdot \vec{n_{z}}$$

where, $\vec{F_{E}}$ is the Coulomb force; $\vec{F_{D}}$ is the dielectrophoretic force; $\vec{F_{I}}$ is the image force; $\vec{F_{V}}$ is the van der Waals force; $\vec{F_{C}}$ is the capillary force; $\vec{F_{G}}$ is the gravitational force; and $\vec{n_{z}}$ is the z‐directional unit vector. The schematic of z‐directional forces is illustrated in Figure S5 (Supporting Information). Among them, only *F_E_* and *F_D_* contribute the upward force to repel the particle from the glass surface. To estimate *F_E_*, the amount of charge carried by the particles is estimated according to the aforementioned particle charging mechanism. Before an electric field is applied, the water film system is assumed to be electrically neutral. At the same time, no charge is injected into the water film system since the particles and glass surface are not in contact with electrodes. Therefore, the total amount of charges carried by the bouncing particles is equal to that by the glass surface, but with opposite polarity. The surface charge density of the particles is roughly assessed as −5.3 µC m^−2^ from the measured results on the glass cover (Figure 3d‐i) with detailed calculation in Note S2 (Supporting Information). Therefore, the z‐direction value of *F_E_* is estimated as 9.9  ×  10^−8^ N (Details in Note S3, Supporting Information). On the other hand, *F_D_* can be estimated based on the distribution of the electric field, and the maximum upward z‐direction value of *F_D_* is 1.9  ×   10^−13^ N (Details in Note S3, Supporting Information), which is much smaller than *F_E_* in this system. This indicates that the particles are charged to generate the strong Coulomb force *F_E_* and repelled by the Coulomb force.

### 2.3 Output Characteristics of REG and the Output Voltage of VMC

**Figure 4:**
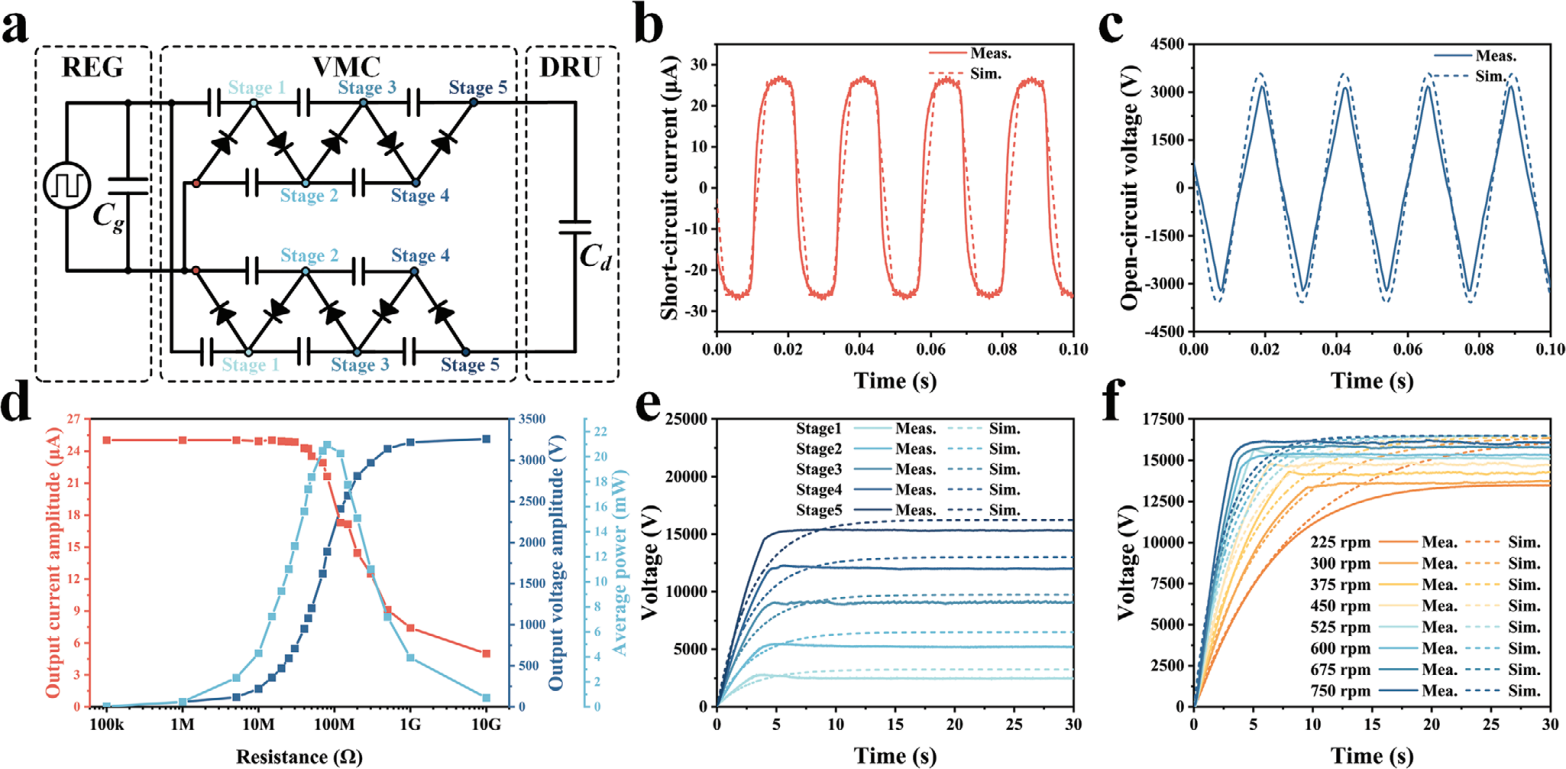
Schematic of the equivalent circuit of the ADRS with simulation and experimental results of the REG and the VMC. a) Schematic of the equivalent circuit. Simulated and measured b) short‐circuit current (*I_sc_*), and c) open‐circuit voltage (*V_oc_*) of the REG. d) Measured amplitude of the output current, voltage, and average power versus resistance. Simulated and measured results of the output voltage of the VMC at e) different stage numbers of the VMC, and f) different rotation speeds of the REG. The results for b–e) are at the rotation rate of 600 rpm.

Although the output current of the electrostatic generator is low, the output voltage is very high, suitable for the dust removal application. The REG can harvest wind energy and generate high‐voltage outputs, and is integrated with a VMC to provide a high‐voltage source (**Figure** **4**a). The REG consists of a patterned charged electret rotator and a stator with a pair of electrode networks to generate electric outputs when there is a relative rotation between the rotator and the stator (Figure S6, Supporting Information), whose equivalent circuit is composed of a current source and a generator capacitor (*C_g_*) connected in parallel.^[^ ^46^ ^]^ The fabricated REG in this work has 4 pairs of electrodes in the stator and a bipolar‐charged rotator with a diameter of 15 cm (Figure S7, Supporting Information), and its *I_sc_* and *V_oc_* are shown in Figure 4b,c, respectively, with the amplitudes of 25.65 µA and 3250 V. Figure 4d shows the amplitude of the output current, voltage, and average power versus resistance, with the maximum average power of 20.98 mW. In addition, the REG has excellent long‐term stability (Figure S8, Supporting Information).

The VMC consists of a network of capacitors and diodes (Figure S9, Supporting Information), and its output terminals are connected with the upper and lower electrodes of the DRU. When the REG starts to work, the output of VMC charges the capacitance (*C_d_*) formed by the DRU. The number of the stage of the VMC determines its output voltage. Here, simulations and experiments are carried out to design the VMC, and the details of the simulation are shown in Note S4 (Supporting Information). Figure 4e displays the measured and simulated output voltages of the VMC under different VMC stages at the rotation speed of 600 rpm. The voltage gradually rises and becomes stable after a short period, and the smaller the stage numbers, the lower the voltage and the shorter the time required for the voltage to reach the steady‐state. When the number of stages is 5, the measured output voltage of VMC can reach higher than 15 kV within 5 s. Here, the VMC doesn't connect to the DRU when measuring. The output voltage of the VMC under different rotation speeds with a stage number of 5 is shown in Figure 4f. The simulated stable voltages tend to be similar to those at different rotation speeds, while the measured one is lower at lower rotation rates due to the leakage current of the diodes and the capacitors. The lower rotation speed results in a longer time for the generator to reach the steady‐state voltage. However, even at a low rotation speed of 150 rpm, the stable voltage of the VMC can reach 13 kV to ensure a high voltage for the dust removal process.

We also simulate the voltage outputs on the DRU with different *C_d_* because different sizes or numbers of solar panels will lead to different *C_d_* values and the simulated results are shown in Figure S10 (Supporting Information). It is observed that the charging speed decreases as the *C_d_* value increases without affecting the stable voltage outputs. Based on these results, the REG with VMC can provide high voltage for DRUs. On the other hand, the REG has high output voltage but very low output current and power such that it won't induce the safety issues of the solar panels or the Joule heating effect. Moreover, it autonomously initiates dust removal when the wind blows, often carrying dust accumulation.

### 2.4 Dust Removal Performance of ADRS and Parameter Optimization of DRU

**Figure 5:**
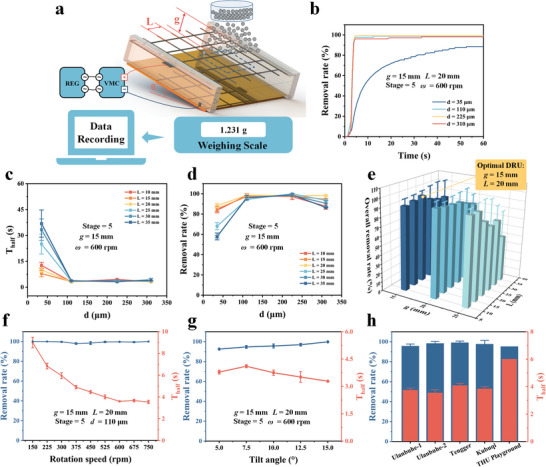
Impact of different parameters on the dust removal effect. a) Schematic of the experimental setup. b) Removal rate versus time for different particle sizes. c) Removal rate and d) *T_half_* versus particle size under different electrode pitch distances (*L)* of the mesh electrode. e) Overall removal rate versus *L* and *g*. f) Removal rate and *T_half_* versus the rotation speed. g) Dust removal rate and *T_half_* under different tilt angles. h) Dust removal rate and *T_half_* for different desert sands. Figure 5g is with Ulanbuhe‐1 Desert sand. Here, the removal rates are all at 60 s. The error bars correspond to the standard deviation (SD) over three tests.

While validating the principles and optimizing parameters, an experimental setup for the dust removal test is established as shown in **Figures** **5**a and S11 (Supporting Information) The DRU used in this experiment consists of an upper mesh electrode and a lower copper plate electrode covered with a thin glass with a tilt angle (*θ*) of 10°. The default values for parameters in the general dust removal test are provided in Table S2 (Supporting Information). Note that the voltage on the DRU is ≈9 kV, lower than the output voltage of the VMC, due to discharge at the tips of the upper mesh electrode, which could be suppressed through insulation sealing of the tips. Silica particles are used as dust in basic experiments, and their scanning electron microscope (SEM) image is shown in Figure S12a (Supporting Information). Initially, dust is dispersed into the DRU using a sieve with a specific mesh size so that ≈1.5 g of dust is evenly distributed on the glass cover. The mass change of the DRU with dust is continuously recorded by a weighting scale during the dust removal process.

To optimize the DRU, the dust removal effects under different dust sizes are tested with different key parameters: REG's speed and desert sand types (Table S3, Supporting Information). The removal rate versus time for different particle sizes is shown in Figure 5b. Figure 5c,d shows the removal rate at 60 s and *T_half_* for different dust particle sizes under different mesh lengths (*L*) of the upper mesh electrode with the stage number of 5 and more detailed data are shown in Figures S13–S16 (Supporting Information). For dust with particle size >100 µm in diameter, the removal effect is almost the same. For the 35 µm in diameter dust, the removal rate is low and it takes a relatively long time to remove 50% dust as small particles result in weak Coulomb force and more time to remove dust fully. Figure 5e shows the overall dust removal rate, which is defined as the average removal rate of the four types of dust with different gaps (*g*) between the upper and lower plate electrodes, and electrode pitch distance, *L*. The overall removal rate tends to decrease as *g* increases. For example, if *g* = 15 mm and *L* = 20 mm, the overall removal rate is the highest for 96% at 60 s. In addition, a large VMC stage number leads to a fast dust removal speed as expected in Figure S17 (Supporting Information). The removal rate at 60 s with a stage number larger than three can achieve ≈100%. Moreover, the rotation speed of the REG does not affect the dust removal rate at 60 s as shown in Figures 5f and S18 (Supporting Information), while a low rotation speed results in more time to remove dust. These results show that the ADRS can work in a wide wind speed range.

To evaluate the dust removal effectiveness on actual sands, Ulanbuhe‐1 Desert sands (Figure S9b, Supporting Information) are used to test the optimal designs of DRU of *g* = 15 mm and *L* = 20 mm under different tilt angles with results shown in Figures 5g and S19 (Supporting Information). It can be seen that the larger the tilt angle, the higher the removal rate, and the removal rate at 60 s reaches 100% at the tilt angle of only 15 degrees, which is smaller than the installation standards of the practical solar panels in most regions worldwide.^[^ ^47^ ^]^ In addition, the dust removal rate at 60 s for sand dust from Ulanbuhe, Tengger, Kubuqi deserts, and Tsinghua playground all exceeded 93%, as depicted in Figures 5h and S20 (Supporting Information).

### 2.5 Wind‐Driven Dust Removal and Test of the Impact of ADRS on Solar Panels

**Figure 6:**
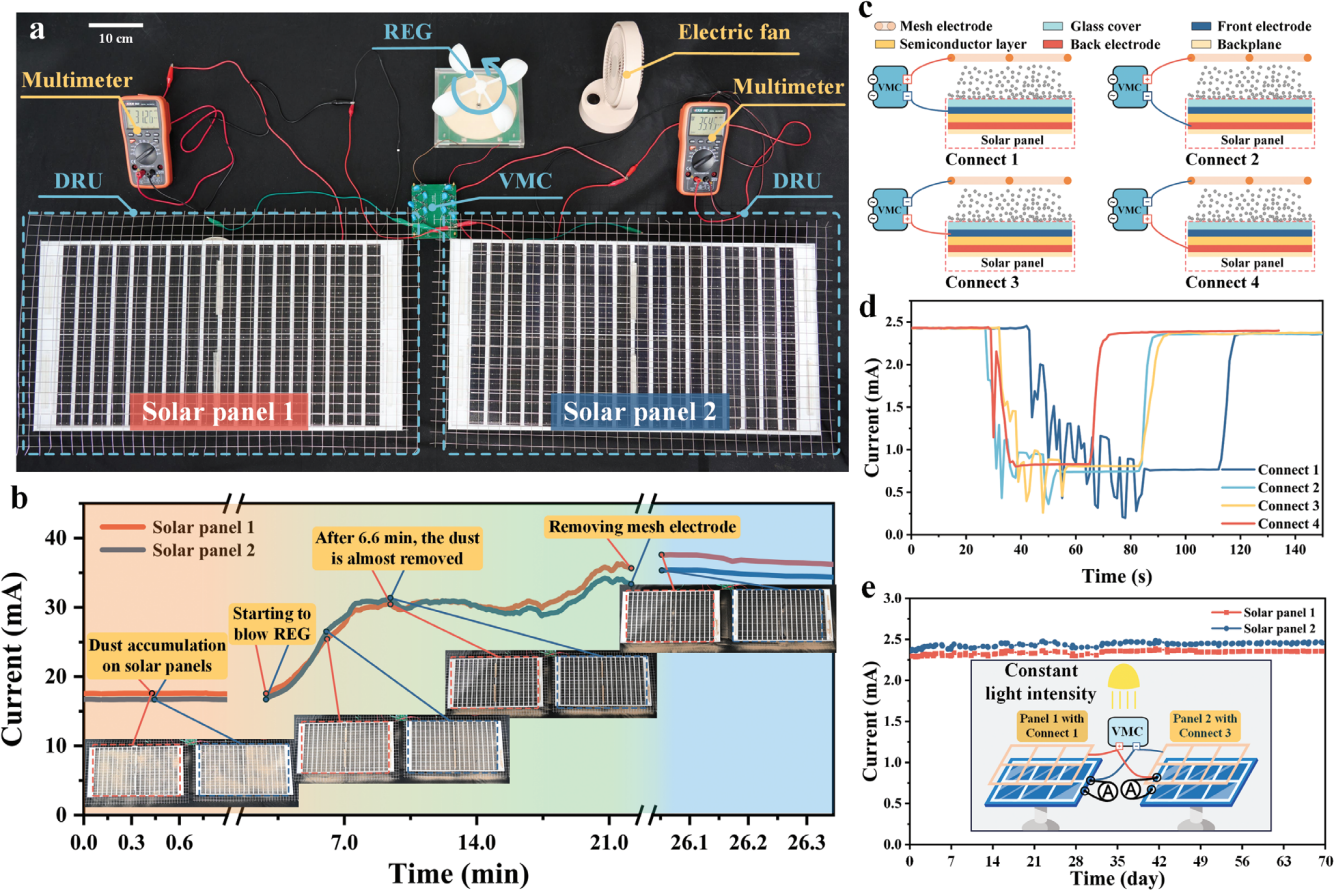
Dust removal for real solar panels and the impact of ADRS on solar panels. a) Photograph of the experimental scene. b) Output currents of two solar panels versus time during the dust removal process. c) Four connection modes, and d) output currents of the solar panel with four connection modes during the dust removal process. e) Output currents of the two solar panels connected to ADRS for long‐term testing.

To verify the feasibility of the ADRS in application environments, dust removal experiments are conducted for real solar panels under the window in a laboratory, with one wind‐driven REG and VMC driving to two DRUs for two solar panels, as shown in **Figure** **6**a. A wind cup is connected to the rotator of the REG as shown in Figure S21a (Supporting Information). Under a wind speed of 1.6 m s^−1^, the REG has a rotation speed of 24 rpm and outputs *I_sc_* of 6.5 µA and a peak‐to‐peak *V_oc_* of 6,780 V as shown in Figure S21b,c (Supporting Information). The REG also exhibits long‐term stability for over 1920000 rotation cycles in Figure S17d. (Supporting Information)

The output currents of the solar panels are measured during the dust removal process as shown in Figure 6b with details in Movie S3 (Supporting Information). Before the REG starts to work, the output currents of the panels with Ulanbuhe‐1 dust are 17.58 and 16.71 mA under slightly different lighting conditions from the window. As REG starts to operate, the output currents of both panels gradually increase and reach 30.61 and 31.33 mA 6.6 min after the dust removal process. Subsequently, the output currents fluctuated as the light condition changed. After removing the mesh electrodes, the output currents increase slightly to 36.28 and 34.42 mA from 35.58 and 33.27 mA, respectively, indicating the mesh electrode blocks ≈2.0% and 3.5% of light, which is generally acceptable in practical applications in desert areas with severe dust accumulation. To evaluate the reliability and sustainability of the ADRS in the real environment, we conducted wind‐driven dust removal experiments on the roof of a building at Tsinghua University, as depicted in Movies S4 and S5 and Note S5 (Supporting Information). The experimental results demonstrate that ADRS also exhibits excellent dust removal performance in the real environment.

It is noteworthy that the positive or negative terminal of VMC and the front or back electrode of the solar panel can be swapped, as depicted in Figure 6c. The output currents of the solar panels with different connection modes from spreading dust to finishing the dust removal process are presented in Figure 6d with experimental details in Note S6 (Supporting Information). The output current of the solar panels fluctuates and decreases during the dust spreading process, and quickly recovers and reaches a stable‐state after the REG starts working to remove the dust. Furthermore, the performance of the solar panel is not affected by applying a high DC voltage between the output electrode of the solar panel and the mesh electrode as experimental results show good stability of two panels connected to ADRS under a constant lighting condition in Note S7 (Supporting Information). The output currents of the two solar panels with different connection modes for 70 days are depicted in Figure 6e, during that the REG is always driven by a motor and a high voltage is applied on the DRU, except for periods when the solar panel current is measured. It can be found that a high voltage on the DRU doesn't affect the performance of the panel with different connection modes. These experimental results illustrate that ADRS has a strong possibility and great flexibility for real applications.

## 3 Discussion

In this work, a good dust removal efficiency has been achieved for particles with a diameter greater than or equal to 35 µm. However, for the particles of 15 µm diameter or smaller, the dust removal efficiency has reduced, as shown in Figure S22a (Supporting Information) as the Coulombic force decreases with respect to the square of the diameter under a constant surface charge density. However, Figure S22b–e and Movie S6 (Supporting Information) show particles of 15 µm diameter are repelled and deposited on the central region of the mesh, indicating that small dust particles could be removed by optimizing the system parameters such as the pitch of the mesh electrodes, the gap between upper and lower electrodes, as well as the applied voltage.

It is noteworthy that although the shade of the mesh electrodes slightly reduces the solar panel outputs, this influence could be decreased by optimization of mesh electrodes. In the current experiments, mesh electrodes are commercial mesh with limited pitch and wire diameter options. To future improve the efficiency, the pitch of the mesh electrode might be arranged according to the wiring arrangement of the solar panel with a thinner wire diameter to minimize the blockage of sunlight. Besides, it can be found that few dust particles settle on the DRU after dust removal, as shown in Figure 1e. Thus, the support structure of the DRU can be designed in a pyramid shape, and the mesh electrodes with the thin wire diameter can be utilized to prevent dust accumulation on the DRU.

In summary, an autonomous dust removal system powered by wind energy has been developed. The ADRS comprises a REG, a VMC, and DRUs. The REG with VMC harvests wind energy to provide a high DC voltage between an upper mesh electrode and one of the output electrodes of the solar panel to generate a strong electrostatic field. This field charges the dust particles on the panel glass cover, assisted by adsorbed water, and repels them with a significant Coulombic force. Experimental results have manifested that the ADRS can work effectively in a wide range of environmental conditions including dust particle size and type, wind speed, humidity, and panel inclination angle. Under the low wind speed of 1.6 m s^−1^, two DRUs for two solar panels driven by one REG and VMC have been shown to remove most dust effectively within a short time of 6.6 min. The high voltage output characteristic of the electret generator perfectly aligns with the requirements for dust removal, while its low current output ensures the safety of solar panels. The system didn't impact the panel performance for long‐term operation. As such, the ADRS has the advantages of low cost, simple structure, high efficiency, and excellent dust removal performances, while it has minimum interferences to the performance of the solar panels for practical applications.

## 4 Experimental Section

### Fabrication of the DRU for the Basic Experiments

The upper mesh electrodes were fabricated by laser‐cutting on thin steel plates of 0.8 mm in thickness to obtain specific mesh electrodes. The lower plate electrode was cut from a copper‐clad plate with an epoxy glass fiber substrate, and covered with a transparent glass layer with a thickness of 0.2 mm. The effective dust removal area of the device was ≈100 × 100 mm.

### Preparation of the Dust Particles

Silica particles with average particle diameters of 35, 110, 225, and 310 µm, CaO, Al_2_O_3_, and Fe_2_O_3_ particles with an average particle diameter of 110 µm were obtained from The Institute of Metal Research, Chinese Academy of Sciences. The real sand dust particles were Ulanbuhe‐1, Ulanbuhe‐2, Tengger, Kubuqi deserts, and Tsinghua playground with an average diameter of ≈150, 110, 95, 150, and 340 µm, respectively. These sand dust particles were placed on the DRU with a sieve with a mesh size of 225 µm.

### Fabrication of the REG

The stator of the REG with electrode networks was fabricated using the PCB process. The rotator with a diameter of 15 cm consists of three layers: the epoxy resin substrate with a thickness of 1.6 mm, the middle layer of a PI double‐sided adhesive tape, and the top layer of a patterned bipolar‐charged polytetrafluoroethylene (PTFE) film with the thickness of 30 µm, which was charged by the patterned contact micro discharge method with −4500 V for 5 min and 4500 V for 3.5 min according to the previous works.^[^ ^48^ ^]^ The rotator was assembled to the axis of a motor for the basic experiments and to the axis of a wind cup for the wind‐driving dust removal test and then assembled with the stator.

### Fabrication of the VMC

Ceramic capacitors with a capacitance of 1 nF and a permissible voltage of 20 kV and diodes with a high reverse breakdown voltage (R5000) were used. To ensure that the breakdown voltage of the diode was high, two Zener diodes were used together in series to achieve the desired voltage.

### Preparation of the Demonstration

The upper mesh electrode of the DRU was cut from a wire netting structure with 20 × 20 mm in individual mesh size (Anping Liangwangqiaojiang Wire Mesh Products Co., Ltd.). The package frame of the REG was made of acrylic, and the wind cup was fabricated by a 3D printer. The solar panel in the demonstration has an area of 67 × 35 cm, a peak power of 30 W, and a peak voltage of 18 V (Shandong Tanyue Internet of Things Technology Co., Ltd.). The solar panel in the test under different connection types and stability under high voltages has an area of 27 × 20 cm, a peak power of 5 W, and a peak voltage of 18 V (Chongqing Aike Electronic Technology Co., Ltd.). The dust used in the demonstration was Ulanbuhe‐1 desert sand.

### Measurement

The bouncing particles were recorded by a high‐speed camera (Phantom V1840, Vision Research Inc., USA) with a modular zoom lens system (Zoom 6000, NAVITAR Inc., USA) at a sample rate of 3300 fps, with an LED flashlight (Mankerlight MK38, CN) as the backlight source. For high‐speed microscopic observation, the mesh electrode was grounded and the lower electrode was connected to a DC power supply (Pintech PA1020, CN). Dust particles were observed by SEM (JEOL, JSM‐7401, JP), and the average particle diameters of the dust particles were obtained by Image J. The mass change was measured and captured with a precision weight scale with an accuracy of 0.001 g and a range of 200 g (LEAPING Co., Ltd.). The relative humidity was measured with hygrometers (ASAIR, AS108D, CN) and (Anymetre, TH802A, CN). The output current and voltage of REG were measured and captured by an oscilloscope (KEYSIGHT DSOX2024A, US) with a homemade circuit. The probe of the oscilloscope with the input parameters of 10 MΩ was used. The output voltage measured with a load resistor of 10 GΩ was regarded as the open‐circuit voltage. The high DC output voltage of the VMC was measured with a homemade voltage division circuit with a division ratio of 4580:1. The output current of the solar panels was measured by a multimeter (VICTOR VC86E, JP). The rotation speed of the REG was calculated according to the frequency of the output voltage captured by the oscilloscope. The wind speed was tested with a handheld anemometer (TRSi TA642B, CN).

## Conflict of Interest

The authors declare no conflict of interest.

## Author Contributions

R.D. conceived the idea, and Z.C. and X.Y. supervised the project. R.D., Y.C., and X.Y. developed the experimental devices. R.D., J.T., and K.D. conducted the simulation and data analysis. R.D., Y.C., X.Q., and X.Y. conducted the measurements and analyzed the experimental data. Z.C., W.Y., Z.W., S.L., and L.L. helped discuss the results and commented on the manuscript. R.D. and X.Y. wrote the paper. All authors contributed to the paper.

## Supporting information

Supporting Information

Supplemental Movie 1

Supplemental Movie 2

Supplemental Movie 3

Supplemental Movie 4

Supplemental Movie 5

Supplemental Movie 6

## Data Availability

The data that support the findings of this study are available from the corresponding author upon reasonable request.
