# Supplementary material for: Self‐Powered Autonomous Electrostatic Dust Removal for Solar Panels by an Electret Generator
Source: Adv Sci (Weinh). 2024 May 5;11(26):2401689. doi: 10.1002/advs.202401689 (PMC11234423; doi:10.1002/advs.202401689)
Supplement: Supplementary file 1 — Supporting Information [file ADVS-11-2401689-s001.pdf]

ADVANCED SCIENCE
Open Access

Supporting Information

for *Adv. Sci.*, DOI 10.1002/advs.202401689

Self-Powered Autonomous Electrostatic Dust Removal for Solar Panels by an Electret Generator

Rong Ding, Zeyuan Cao, Junchi Teng, Yujia Cao, Xiaoyu Qian, Wei Yue, Xiangzhu Yuan, Kang Deng, Zibo Wu, Shuiqing Li, Liwei Lin and Xiongying Ye**

Supplementary Materials for

Self-Powered Autonomous Electrostatic Dust Removal for Solar Panels by an Electret Generator

Rong Ding,[1] Zeyuan Cao,[1] Junchi Teng,[1] Yujia Cao,[1] Xiaoyu Qian,[2] Wei Yue,[3] Xiangzhu Yuan,[1] Kang Deng,[1] Zibo Wu,[1] Shuiqing Li,[2] Liwei Lin[3] and Xiongying Ye[1]**

[1]State Key Laboratory of Precision Measurement Technology and Instruments, Department of Precision Instrument, Tsinghua University, Beijing 100084, China

[2]Key Laboratory for Thermal Science and Power Engineering of Ministry of Education, Department of Energy and Power Engineering, Tsinghua University, Beijing 100084, China

[3]Berkeley Sensor and Actuator Center and Department of Mechanical Engineering, University of California at Berkeley, Berkeley, CA, 94720, USA

Corresponding Author

*Email: caozy@mail.tsinghua.edu.cn, xyye@mail.tsinghua.edu.cn

This file includes:

Figs. S1 to S29
Tables S1 to S5
Supplementary Note S1 to S7
References (S1 to S2)
Legends for Movies S1 to S6

Other Supplementary Materials for this manuscript include the following:

Movies S1 to S6

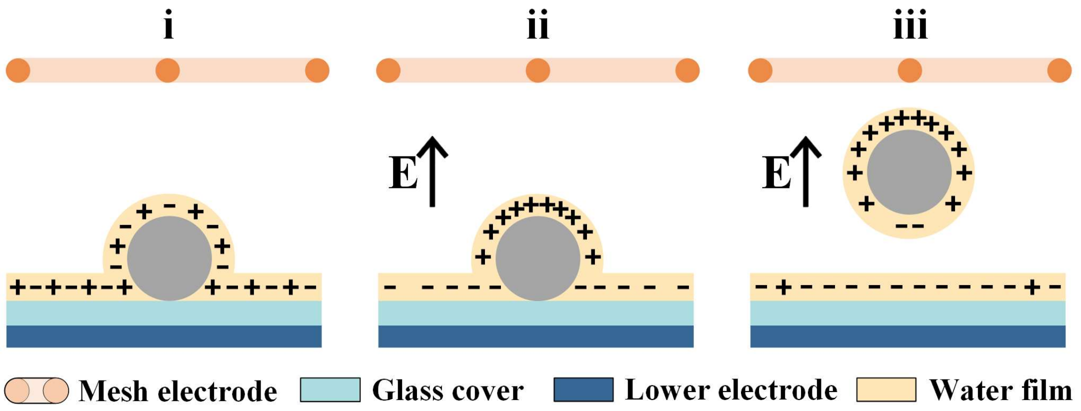

Fig. S1 Charging process of a dust particle on a glass cover under an electric field. An upward electric field is applied, and the particle accumulates positive charges.

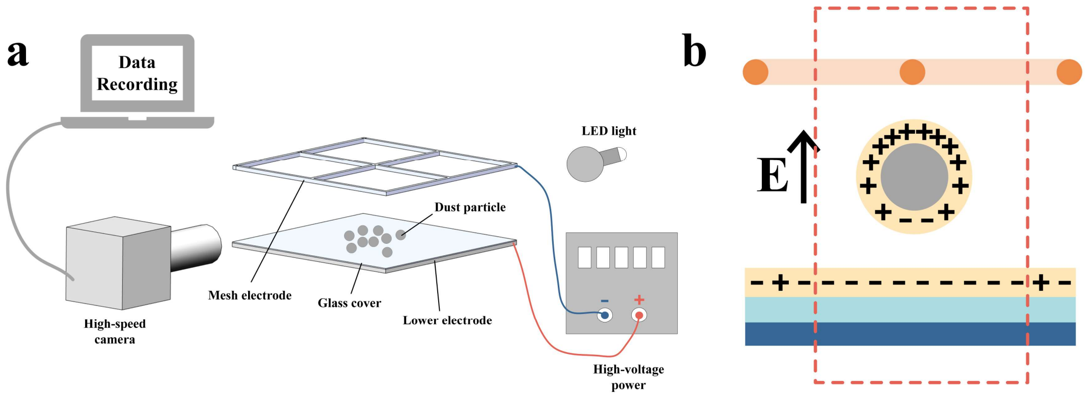

Fig. S2 Schematic diagram of the experimental setup and area for high-speed microscopic observation. (a) Schematic of the experimental setup and (b) the area for microscopic observing. The DRU is placed horizontally without any inclination. High voltage is applied to the DRU using a commercial power supply with a voltage value of 4.5 kV. The trajectories of the dust particles between the electrodes are recorded with a high-speed microscopic camera. The dimensions of the DRU used in the actual experiment may be not scaled with the diagram in Fig. 2b. The gap between the mesh electrode and the lower electrode is 10 mm, and the mesh electrode pitch distance is 10 mm.

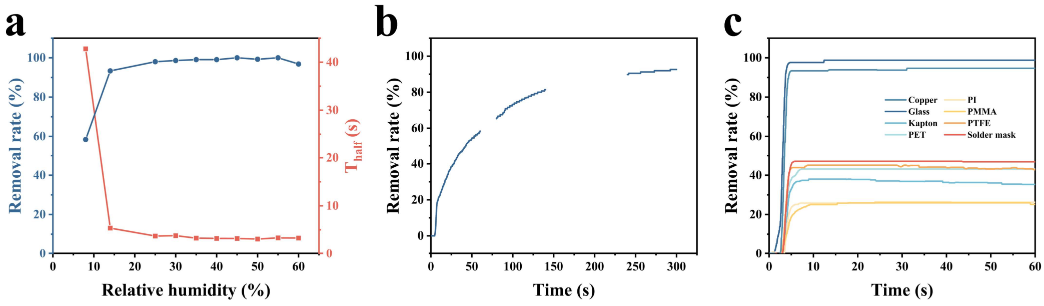

Fig. S3 Dust removal effect under different relative humidity and surface cover materials. (a) Removal rate and T_{half} *versus* relative humidity. (b) Removal rate *versus time when* relative humidity is 8%. (c) Removal rate *versus* time under the lower electrode (copper) and various surface materials covering the lower electrode. In Fig. S3b, the total time of dust removal was 300 s, with each data collection lasting 60 s, and there were intervals between 3 collections.

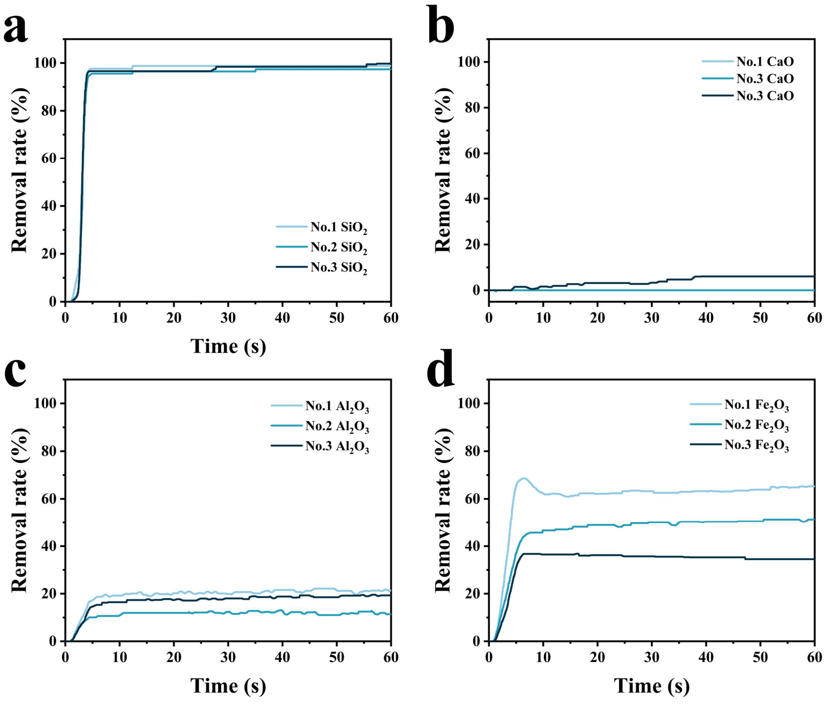

Fig. S4 Dust removal rate *versus* time under different particle materials. The particle materials are (a) SiO_2, (b) CaO, (c) Al_2O_3 and (d) Fe_2O_3.

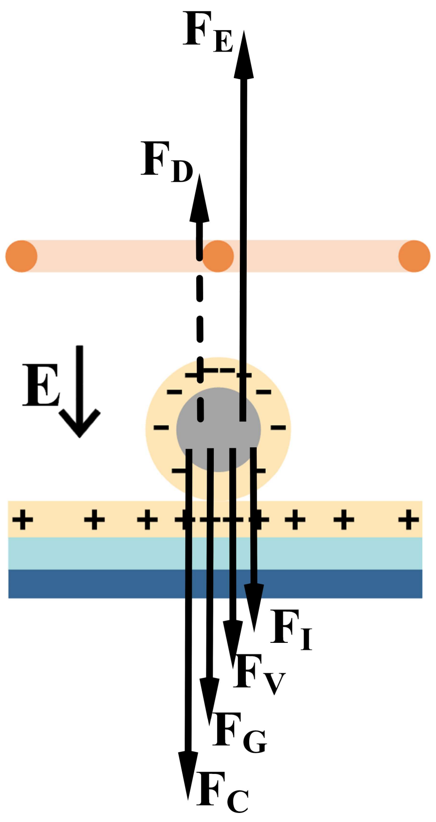

Fig. S5 Illustration of the z-directional forces acting on a particle resting on the glass surface. The direction of F_D could be upward or downward depending on the position.

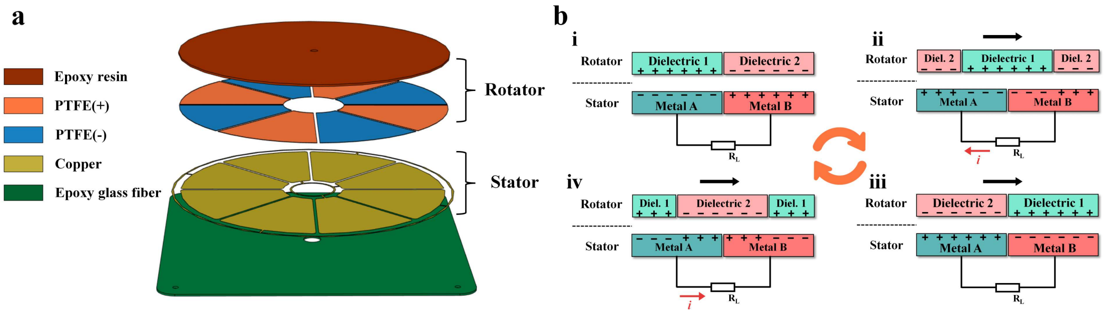

Fig. S6 Schematics of the structure and working principle of the REG. (a) Structure. (b) Working principle.

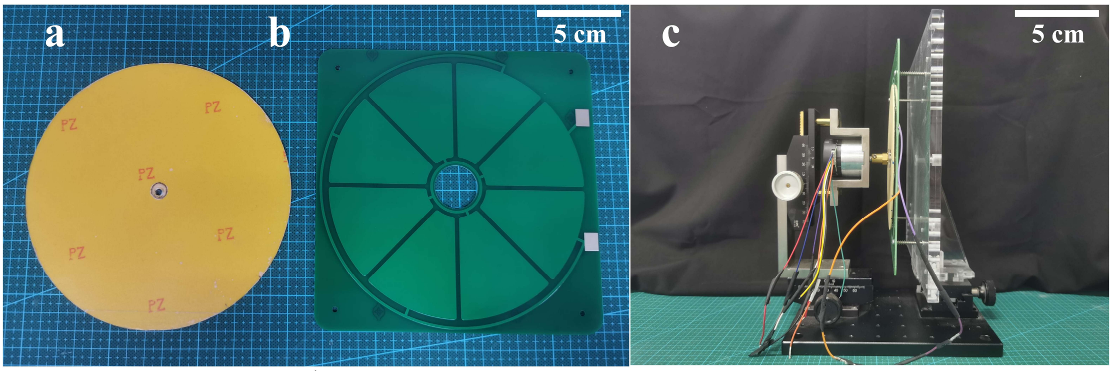

Fig. S7 Photographs of (a) rotator, (b) stator and (c) the REG driven with a linger motor in the experiment system.

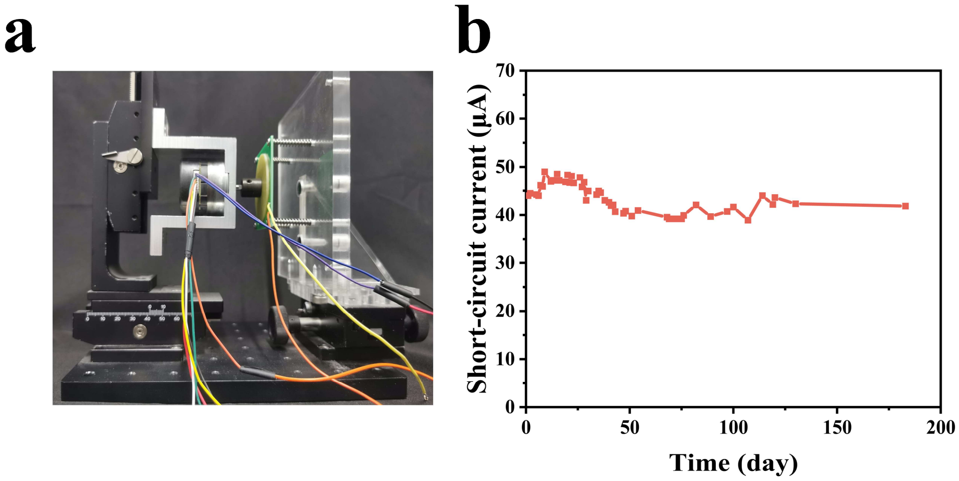

Fig. S8 Experiment of long-term stability of the REG. (a) Photograph of the experimental setup. (b) Long-term stability of the short-circuit current. The REG is with a diameter of 5 mm and pairs of 12. The rotation speed of the REG is 750 rpm in the test. The short-circuit current is measured once in one day. And the result depicts that the charge density of the REG maintains stability for 183 days.

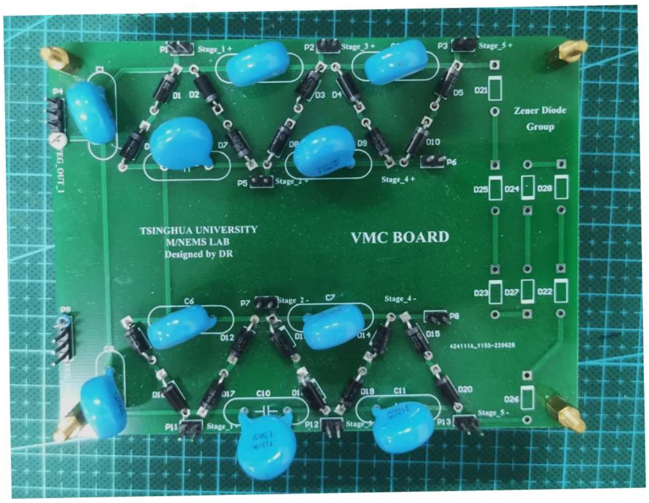

Fig. S9 Photograph of the VMC.

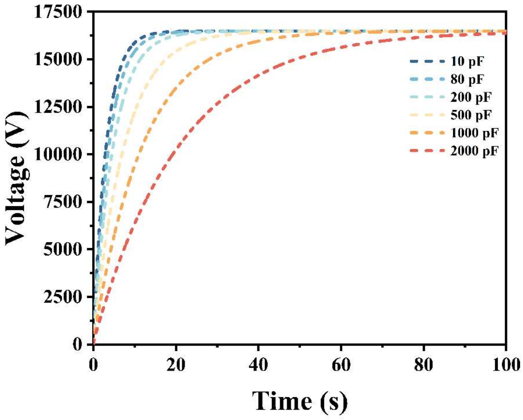

Fig. S10 Simulated voltage on C_d with different C_d.

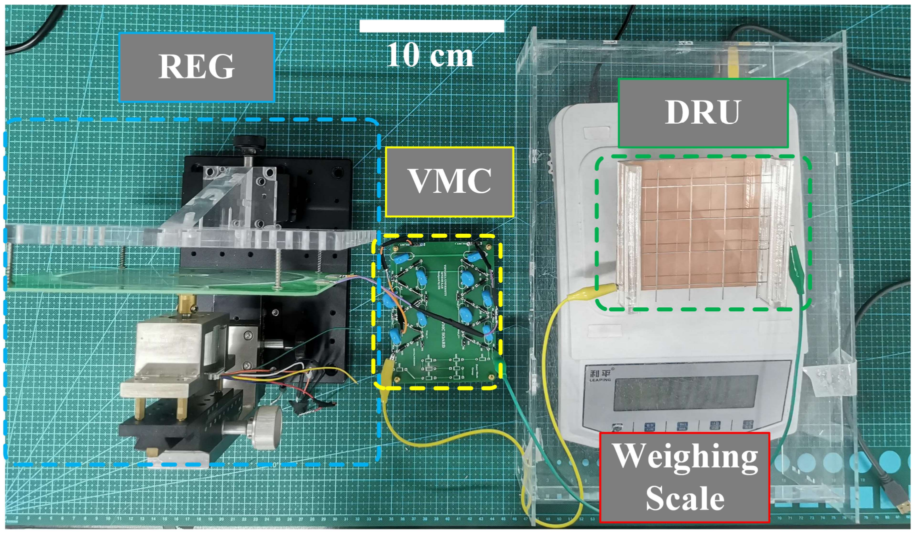

Fig. S11 Photograph of the experimental setup for dust removal test.

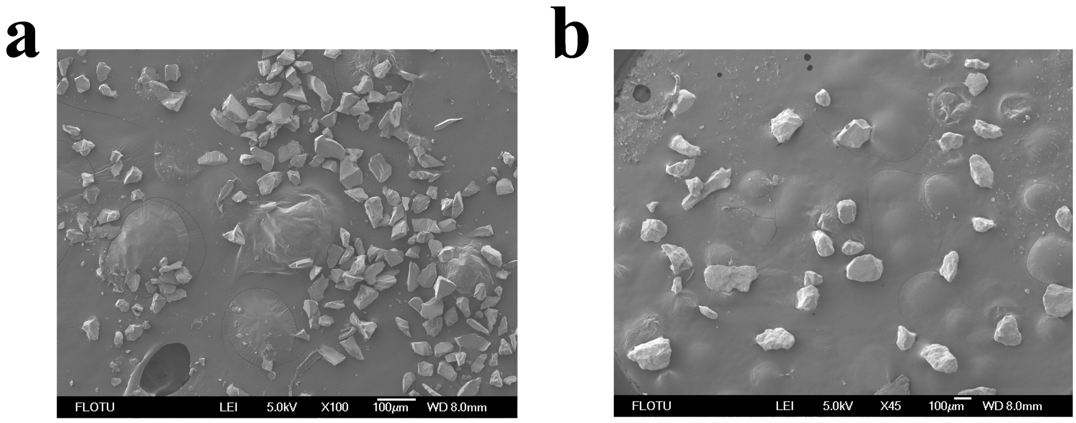

Fig. S12 SEM photographs of the particles. (a) Silica particles with an average size of 35 μm. (b) Ulanbuhe-1 Desert sand particles whose average size is about 150 μm.

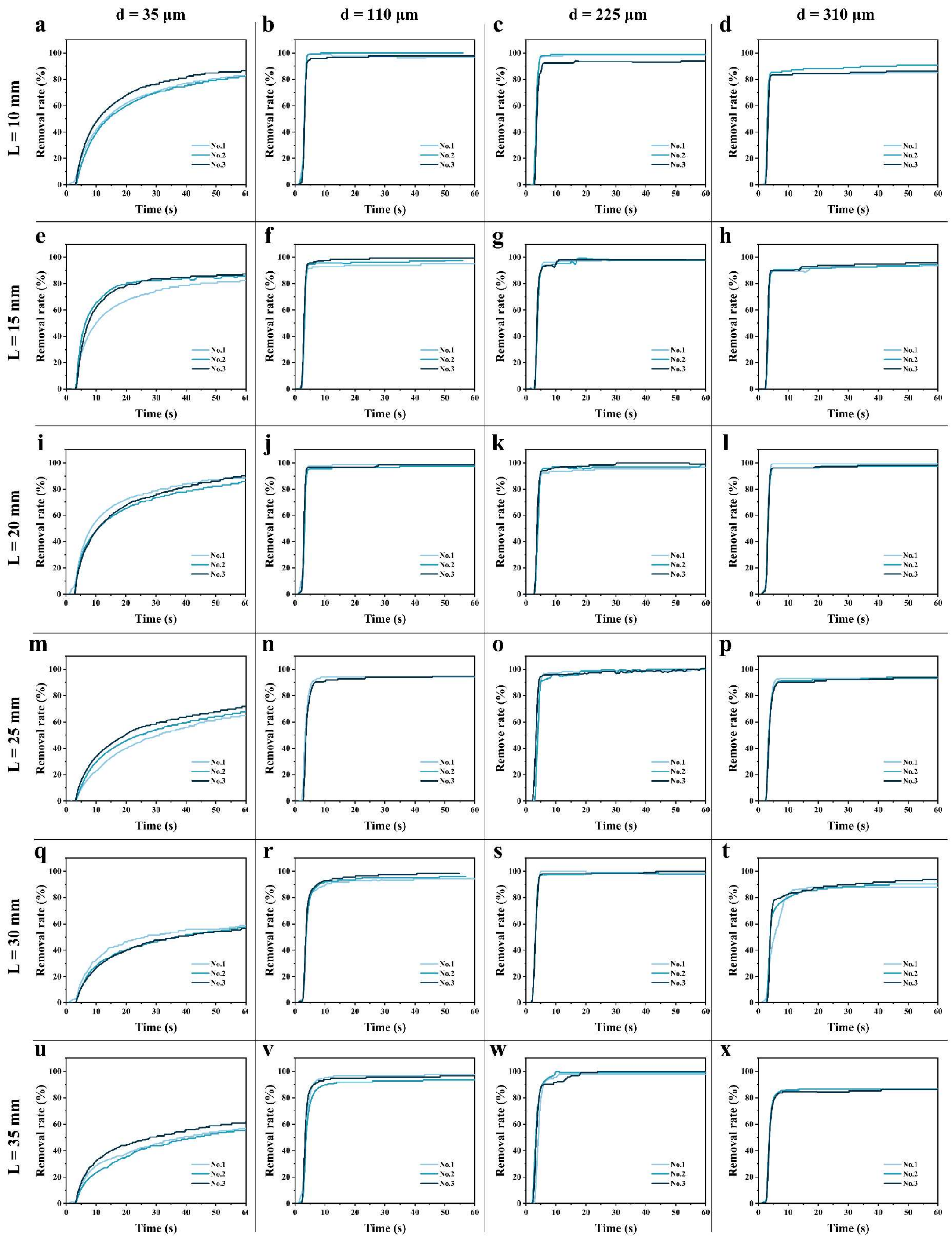

Fig. S13 Results of the removal rate *versus* time at different *L* and particle sizes. g = 15 mm, stage = 5, ω = 600 rpm.

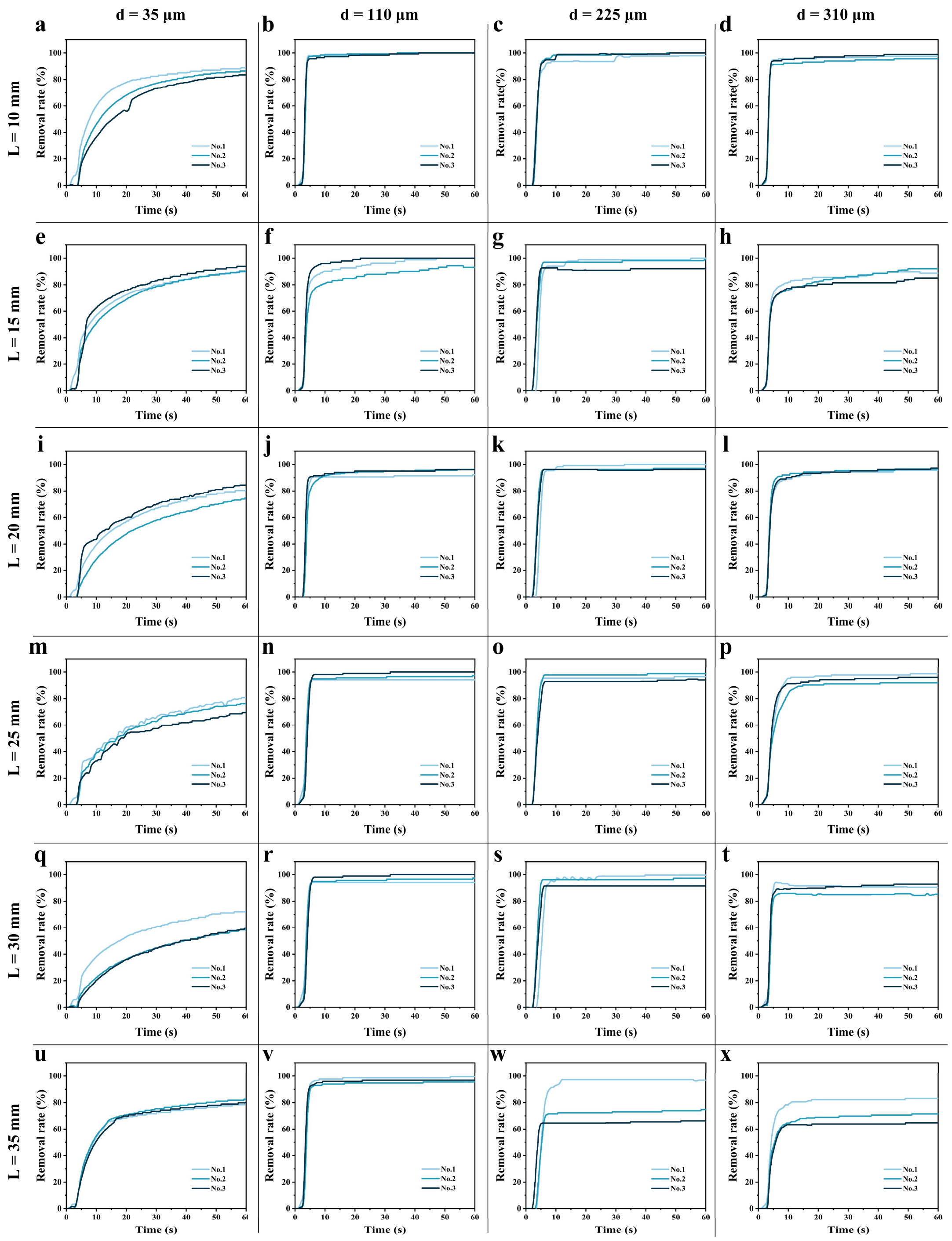

Fig. S14 Results of the removal rate *versus* time at different *L* and particle sizes. *g* = 20 mm, stage = 5, *ω* = 600 rpm.

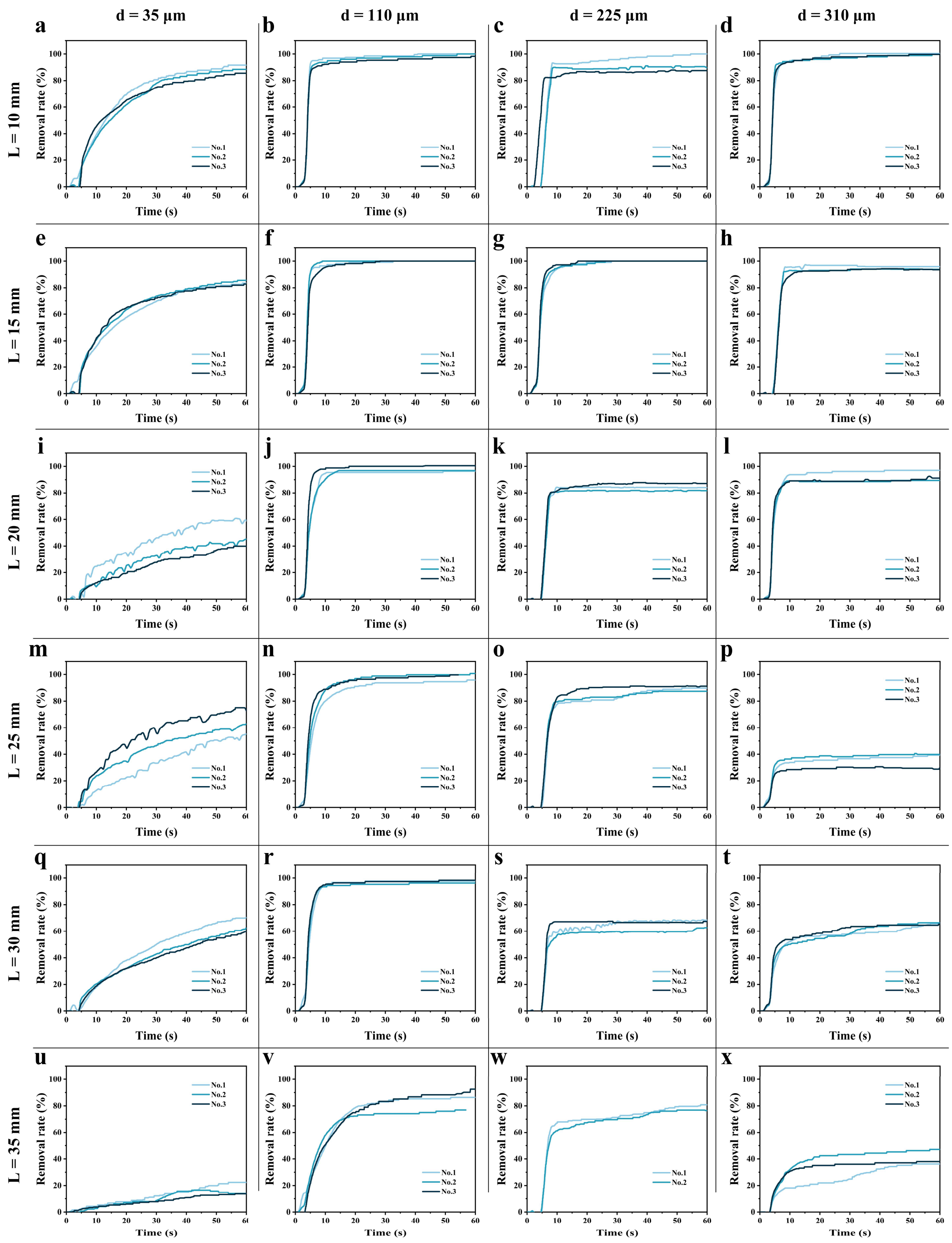

Fig. S15 Results of the removal rate *versus* time at different *L* and particle sizes. *g* = 25 mm, stage = 5, *ω* = 600 rpm.

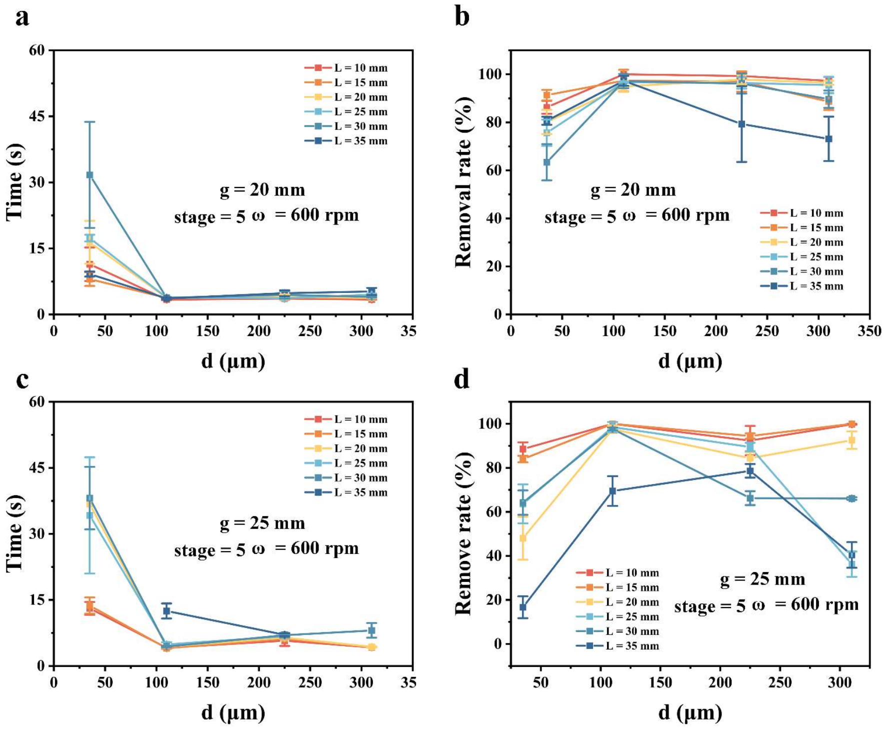

Fig. S16 Summarization of the dust removal effect at different *L* and particle sizes for *g* =20, 25 mm. T_{half} under (a) g = 20 mm and (c) g = 25 mm. Removal rate under (b) g = 20 mm and (d) g = 25 mm. Partial time point data in **Fig. S16c** is missing because the dust removal rate did not reach 50% within 60 seconds.

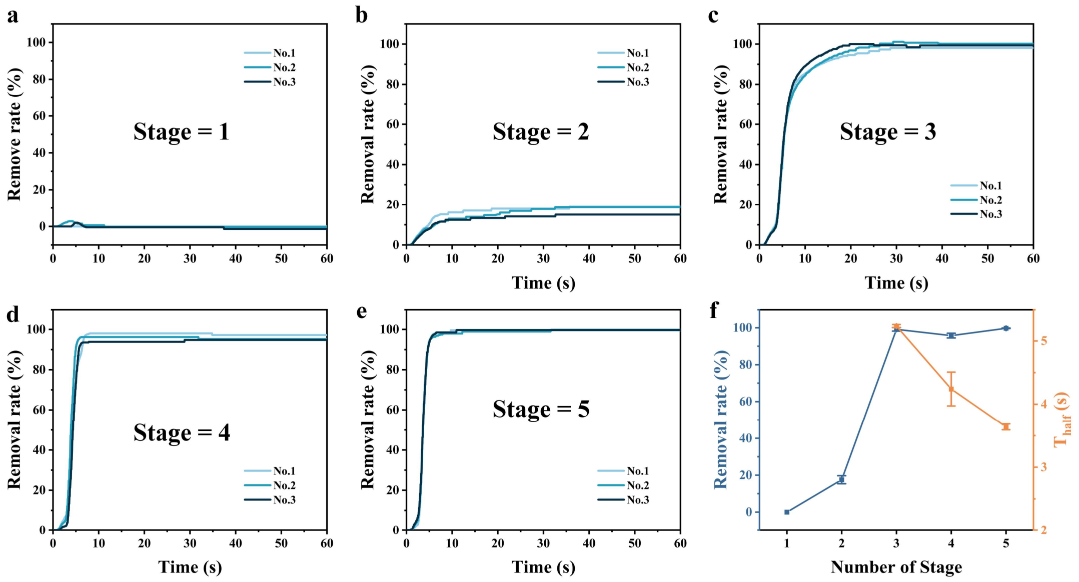

Fig. S17 Results of the dust removal effect at different VMC stage numbers. g = 25 mm, L= 20 mm, ω = 600 rpm, d = 110 μm. Partial time point data in **Fig. S17f** is

missing because the dust removal rate did not reach 50% within 60 seconds.

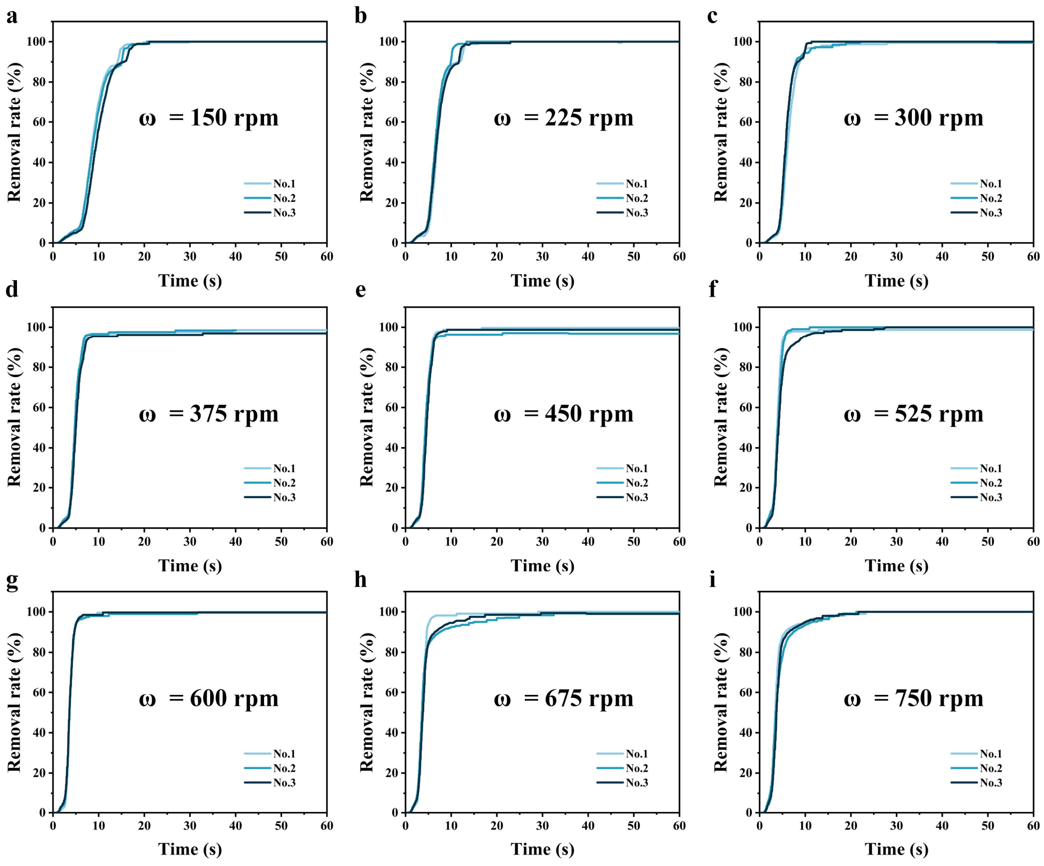

Fig. S18 Results of the dust removal effect at different rotation speeds. g = 25 mm, L= 20 mm, stage = 5, d = 110 µm.

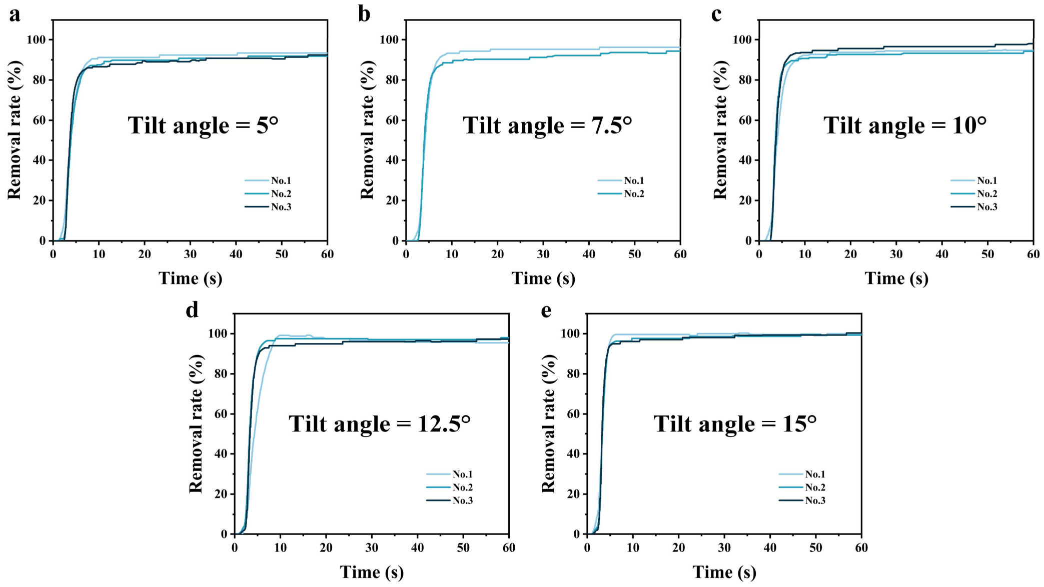

Fig. S19 Results of the dust removal effect at different tilt angles. g = 25 mm, L= 20 mm, stage = 5, ω = 600 rpm, Ulanbuhe-1 desert sand.

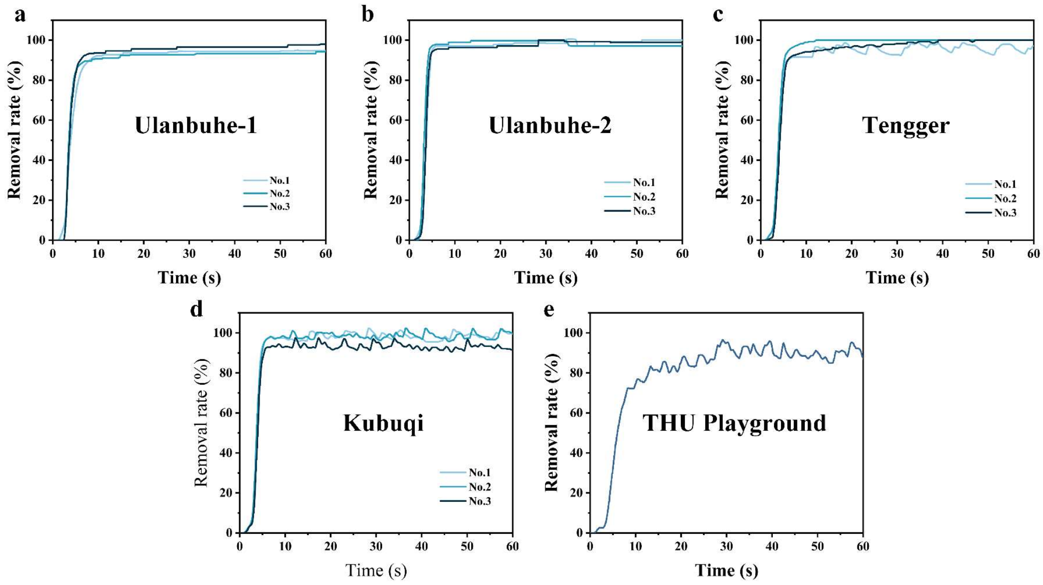

Fig. S20 Results of the dust removal effect with different sand dust. g = 15 mm, L= 20 mm, stage = 5, ω = 600 rpm.

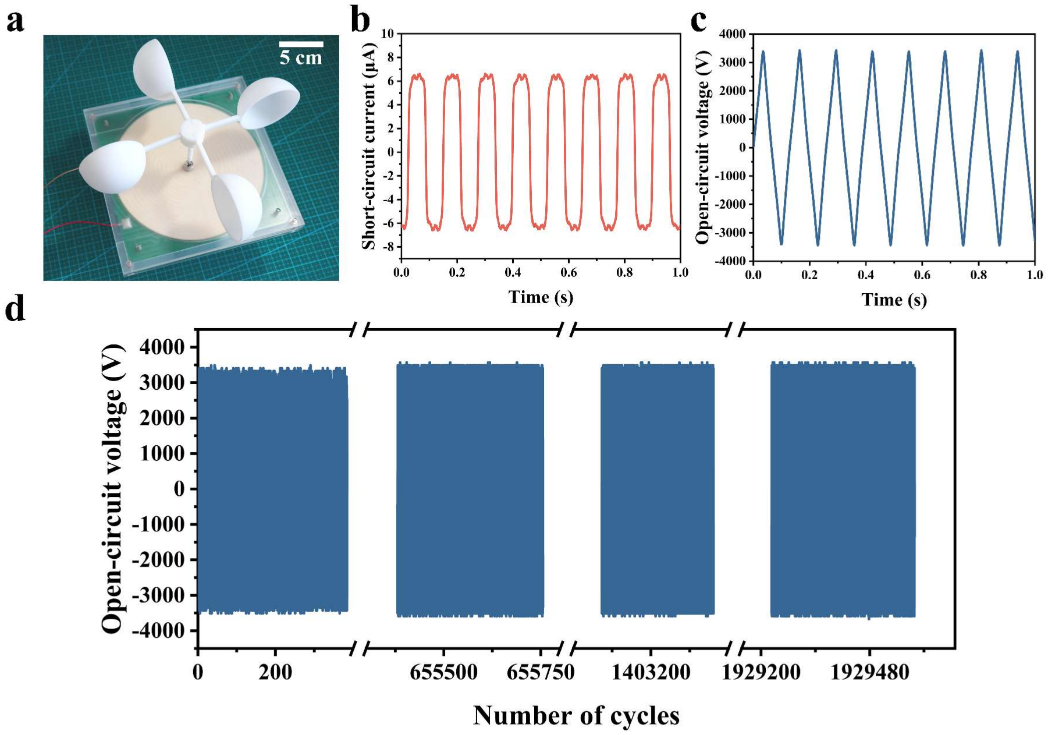

Fig. S21 Structure and output characteristic of the wind-driven REG. (a) Photograph of the wind-driven REG and (b) its I_{sc}, (c) V_{oc} and (e) long-term stability.

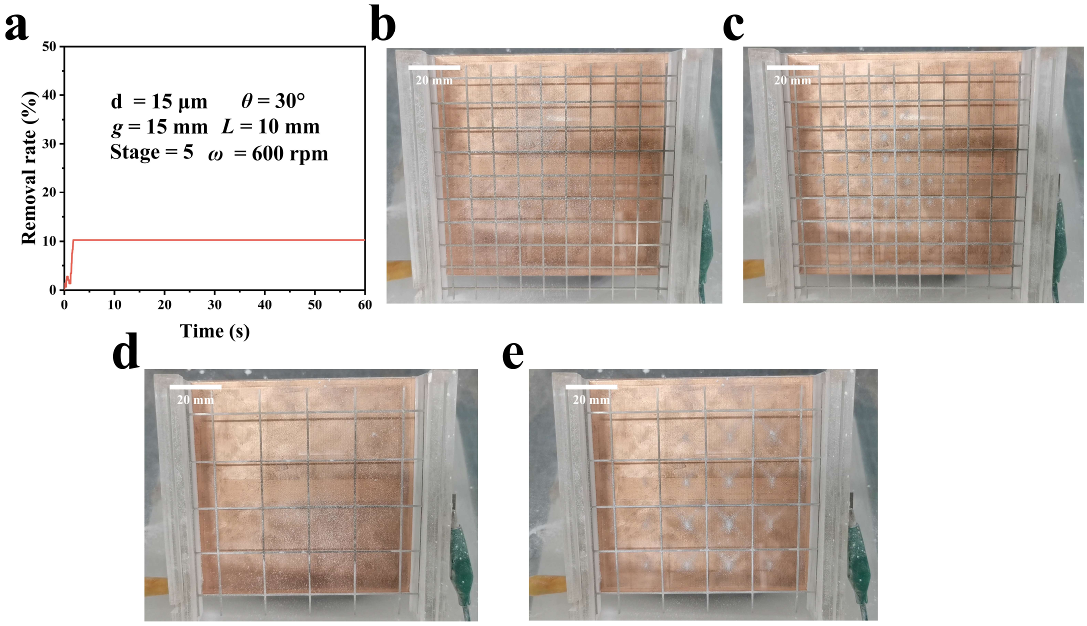

Fig. S22 Results of the dust removal performance for the particles of 15 μm diameter. (a) Removal rate *versus* time. When L = 10 mm, the photographs of DRU (b) before and (c) after the dust removal. When L = 20 mm, the photographs of DRU (d) before and (e) after the dust removal.

Table S1 Parameters and results of the measurement of the surface potential on the glass cover

Parameters of the measurement							
Voltage on DRU when ADRS operated (kV)				~ 9			
Duration of ADRS operation (s)				~ 30			
L (mesh electrode pitch distance, mm)				20			
g (gap between the mesh electrode and the glass cover, mm)				15			
m (mass of the dust particle, g)				~ 1.5			
d (diameter of the dust particle, μm)				110			
Duration of the preparation for measurement (s)				~ 15			
Results of the measurement							
Condition		Without dust			with dust		
Number		1	2	3	1	2	3
Test 1	V_0/V	3	3	3	-10	7	14
	V_1/V	13	11	14	130	110	105
	V_{diff}/V	10	8	11	140	103	91
Test 2	V_0/V	3	-3	-5	2	2	-3
	V_1/V	-8	-16	-30	-125	-130	-160
	V_{diff}/V	-11	-13	-25	-127	-132	-157

Table S2 Default values of the parameters on the general dust removal test

Parameter		Value
DRU	θ (tilt angle, °)	10
	g (gap between the upper and lower electrode, mm)	15
	L (mesh electrode pitch distance, mm)	20
	Surface cover material on the lower electrode	Glass
	h (thickness of the glass cover, mm)	0.2
	Voltage on the DRU (kV) *	~9
Dust	d (diameter, μm)	110
	Particle type	Silica
REG	ω (rotation speed, rpm)	600
VMC	Number of stages	5
Humidity	R_H/%	~ 30
Time	Time of dust removal (s)	60

* The voltage on the DRU, namely, on C_d is measured by a homemade voltage division circuit. Note that the voltage on the DRU is approximately 9 kV, lower than the output voltage of the VMC, due to discharge at the tips of the upper mesh electrode.

Table S3 Parameters of REG, DRU and dust particles on the dust removal test

Parameter		Value								
DRU	θ/°	5	7.5	10	12.5	15				
	g/mm	15	20	25						
	L/mm	10	15	20	25	30	35			
Dust	d/μm	35	110	225	310					
REG	ω/rpm	150	225	300	375	450	525	600	675	750

Note S1: Measurement of the surface potential of the glass cover and calculation of the change of the charge on the glass cover

An electrostatic voltmeter (Trek 347, US) is utilized to measure the surface potential on the glass cover, then the charge is calculated according to the electric potential. The testing procedure is illustrated in **Fig. S23**, in which the mesh electrode connects to positive voltage and particles are negatively charged (Test 1); for opposite charging, just replace the connecting terminals (Test 2). And the photograph of the surface potential measurement setup is shown in **Fig. S24**. To eliminate the influence of the initial potential on the glass surface, we conducted tests under two conditions: without dust (**Fig. S23a**) and, with dust (**Fig. S23b**) on the glass surface. The surface potential is measured before and after the ADRS operation, which is V_0 and V_1, respectively. And the ADRS is operated for 30 s, and then the measurement preparation after the ADRS operation takes about 15 s. The voltage on the DRU is about 9 kV, and each test is repeated 3 times.

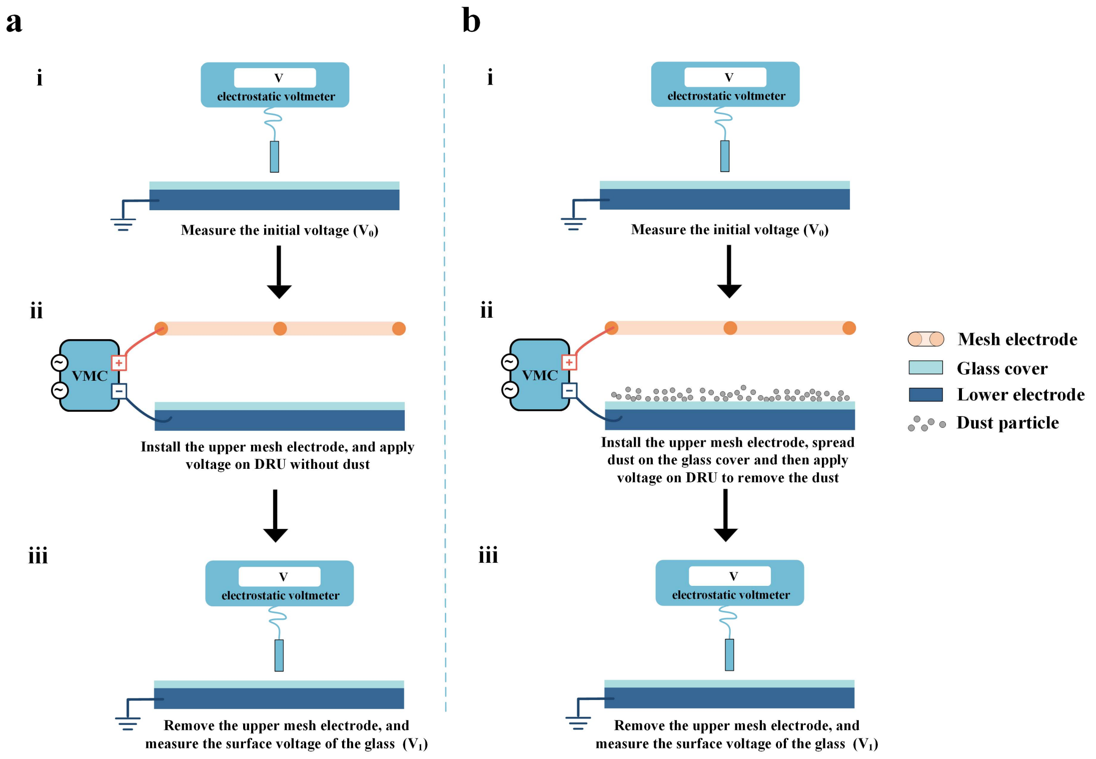

Fig. S23 Experimental procedure of the electric potential of the glass cover before

and after the ADRS operation. (a) without dust and (b) with dust. Here, the mesh electrode connects to the positive voltage.

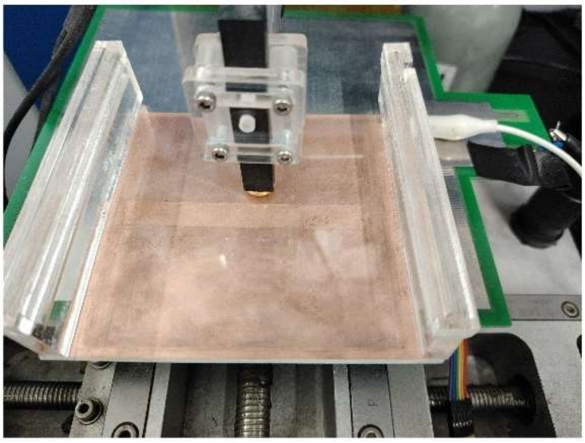

Fig. S24 Photograph of the experimental setup for measuring the surface potential of the glass cover.

Based on the results, we can obtain the change in the surface potential on the glass cover before and after the ADRS operation, denoted as $V_{diff} = V_1 - V_0$. Charge on the glass surface Q_g can be calculated using the following formula:

$$Q_g = \frac{\varepsilon_0 \varepsilon_g S V_{diff}}{h} \qquad (S1)$$

where ε_0 and ε_g are vacuum permittivity and the relative permittivity of the glass, respectively. $\varepsilon_0 = 8.854 \times 10^{-12}$ F/m and $\varepsilon_g = 3.9$. S and h is the area and thickness of the glass cover, respectively, and $S = 0.01\, m^2$ and $h = 0.2\, mm$.

Note S2: Estimation of the surface charge on a particle according to the surface charge of the glass cover.

According to the dust charging mechanism, the total amount of charge carried by the particles is equal to that by the glass surface, but with opposite polarity. From **Fig. 3d-i,iii**, the charge carried by the glass surface, namely, the charge carried by the particles (Q) after dust removal is 192.1 nC and -239.3 nC, respectively.

The mass of one particle:

$$\mathrm{m_p} = \frac{\pi\rho_p d_p^3}{6} \qquad (S2)$$

Here, ρ_p is the density of the particle, and d_p is the diameter of the particle.

The total mass of the particles (M) is about 1.5 g.

Thus, the average charge per particle Q_p can be given by:

$$Q_p = \frac{Q\mathrm{m_p}}{M} \qquad (S3)$$

And the surface charge density of the particle σ_p is:

$$\sigma_p = Q_p/\pi{d_p}^2 \qquad (S4)$$

Therefore, the average charge per particle is -2.0 × 10^{-13} C and 2.4 × 10^{-13} C and the surface charge density σ_p is $-5.3\,\mu C/m^2$ and $6.3\,\mu C/m^2$ when the voltage on DRU is about 9 kV. Due to the duration of the preparation for the surface voltage test is about 15 s and the rapid dispersion of charges, the estimation results of the particle charge by this method are lower than the real.

Note S3: The estimation of the Coulombic force and dielectrophoretic force

The Coulombic force $\overrightarrow{F_E}$ can be calculated as:

$$\overrightarrow{F_E} = \vec{E}Q_p \qquad (S5)$$

We use Comsol Multiphysics to simulate the electric field $\vec{E}$ with a 3D model as shown in **Fig. S25**. The upper electrode is the mesh electrode. The lower electrode is a copper plate and is covered with a thin glass plate. The silica particles are assumed to be spherical. The parameters in the simulation are shown in **Table S4**, which are the same as the parameters in the experimental test in **Fig. 3d-i**. The simulated results of the electric field are shown in **Fig. S26**.

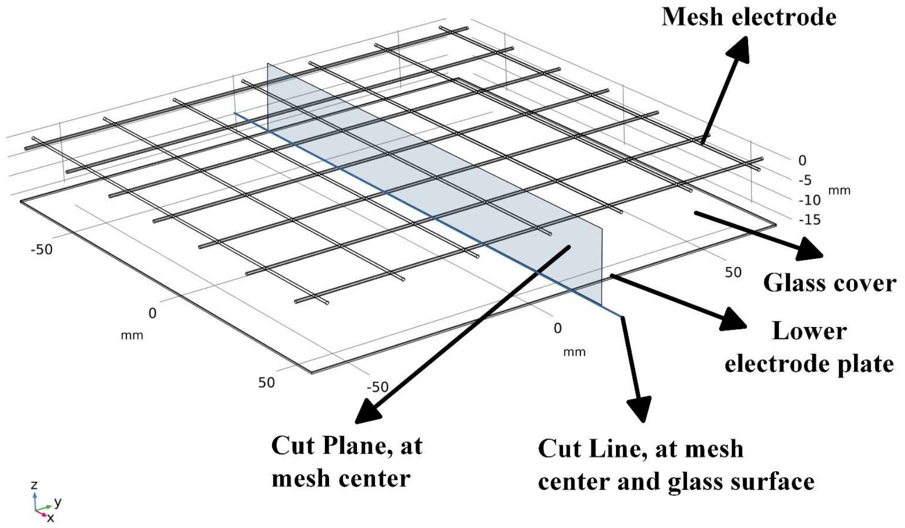

Fig. S25 (a) Schematic diagram of DRU structure in the simulation.

Table S4 Parameters in simulation and calculation

Upper mesh electrode	diameter (mm)	0.8
	mesh electrode pitch distance (mm)	20
Lower plate electrode	thickness (mm)	0.35
	material	copper
gap between the upper and lower electrode (mm)		15
Glass layer	thickness (mm)	0.2
	relative permittivity	3.9
Dust particle	diameter (μm)	110
	density (kg/m^3)	2200
	relative permittivity	3.9
voltage on the upper mesh electrode (kV)		9
voltage on the lower plate electrode (kV)		0
ε_0 (vacuum permittivity, F/m)		$8.854 * 10^{-12}$

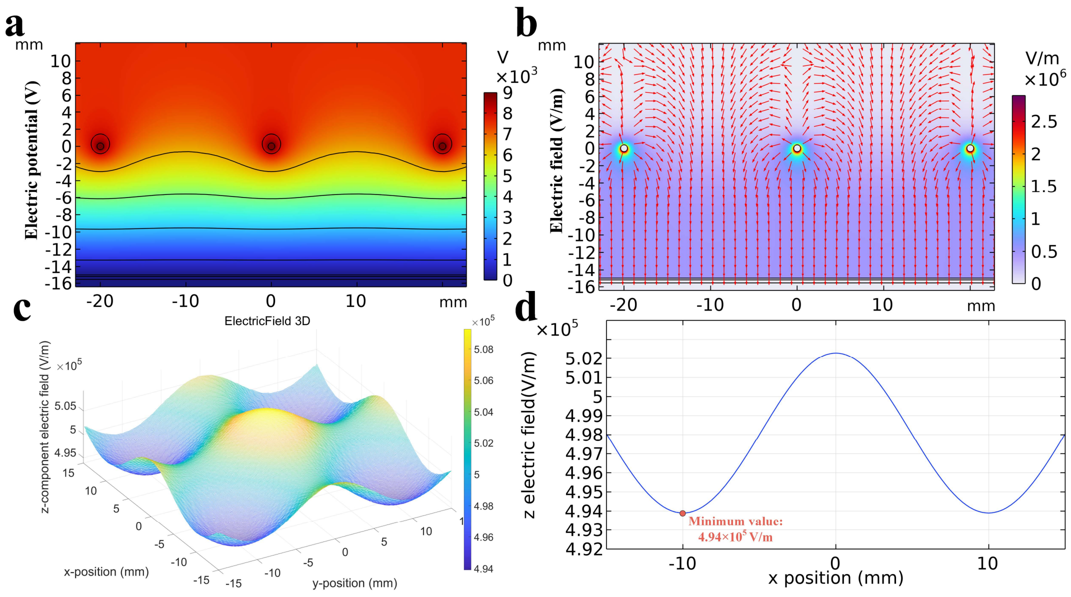

Fig. S26 Distribution of (a) the electric potential and (b) the electric field at the Cut Plane. (c) Distribution of the value of the z-component electric field at the surface of the glass cover. (d) Value of z-component electric field at the Cut Line.

The minimum value of the z-component electric field is at the center of the mesh at the glass surface, which has a value of 4.94×10^5 V/m. From Note S2, the charge carried by a dust particle is $Q_p = -2.0 \times 10^{-13}$ C . The minimum z-direction value of F_E acted on a particle is 9.9×10^{-8} N.

We only consider the first-order dielectrophoretic force,[S1] which can be given by:

$$\overrightarrow{F_D} = (\vec{p} \cdot \nabla)\vec{E} = 2\pi r_p^2 \varepsilon_m K \nabla(\vec{E} \cdot \vec{E}) \qquad (S6)$$

where $\vec{p}$ is the equivalent dipole moment, $\nabla\vec{E}$ is the electric field gradient, r_p is the radius of the particle, and K is the real part of the complex Clausius–Mossotti (CM) factor, which is given by $K = \frac{\varepsilon_p - \varepsilon_m}{\varepsilon_p + 2\varepsilon_m}$, where ε_p and ε_m are the relative permittivity of the particle and the medium, respectively. Substituting the particle radius $r_p = 55\ \mu m$, the relative permittivity of the particle and air $\varepsilon_p = 3.9$, $\varepsilon_m = 1$, based on the simulated electric field distribution, the z-direction value of F_D was obtained, as shown in **Fig. S27**. The approximate maximum upward z-direction value of F_D on the

glass surface is 1.9×10^{-13} N.

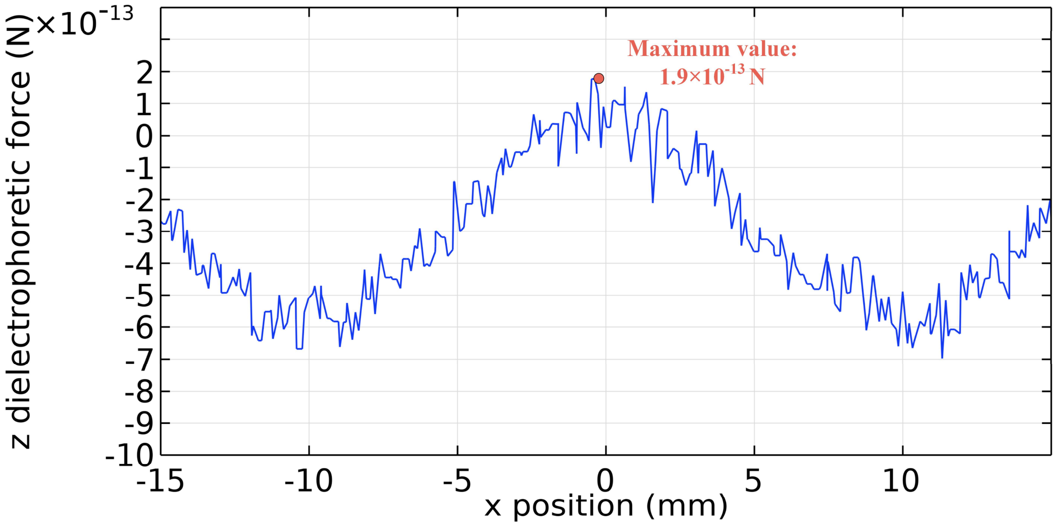

Fig. S27 Z-direction dielectrophoretic force at the Cut Line.

Note S4: Simulation of the outputs of REG and VMC

According to our previous work [S2], the equivalent circuit of an ideal REG can be considered as a square wave current source I_S parallelly connected with the generator capacitor C_g as shown in **Fig. 4a**. The amplitude and period T of I_s equal to those of the short-circuit current I_{sc} of REG. I_{sc} can be expressed as follows under the ideal conditions:

$$I_{SC} = \begin{cases} \frac{1}{2}\sigma(r^2 - r_0^2)n\omega, & t \in [0, \frac{\pi}{n\omega}) \\ -\frac{1}{2}\sigma(r^2 - r_0^2)n\omega, & t \in [\frac{\pi}{n\omega}, \frac{2\pi}{n\omega}) \end{cases} \quad (S7)$$

And the open-circuit voltage V_{oc} can be expressed as follows under the ideal condition:

$$V_{OC} = \begin{cases} \frac{\sigma(r^2 - r_0^2)}{4C_g}(2n\omega t - \pi), & t \in [0, \frac{\pi}{n\omega}) \\ -\frac{\sigma(r^2 - r_0^2)}{4C_g}(2n\omega t - 3\pi), & t \in [\frac{\pi}{n\omega}, \frac{2\pi}{n\omega}) \end{cases} \quad (S8)$$

where σ is twice the charge density on the electret for bipolar charged electret.

Due to the fringe effect of the electric field, the waveform of I_{sc} will be like a trapezoidal wave in the actual case, as shown in **Fig. S28a**. A parameter named *MR* can be defined to quantitatively describe the degree of the fringe effect [S3], and is

expressed as:

$$MR = \frac{T_{rise} + T_{fall}}{T} \qquad (S9)$$

When connecting the VMC, the amplitude of the output voltage of the REG is much lower than that of V_{oc}, as shown in **Fig. S28b**, due to the series of capacitors and diodes will introduce a parallel capacitor (C_{PV}) connected to the generator, resulting in a decrease in V_{oc}. Then the modified electrical model of the ADRS is used in the simulation, as shown in **Fig. S28c**. The parameters used in the simulation are listed in **Table S5**. According to our previous work [S2], the amplitude of I_s is proportional to the rotation rate, and T is inversely proportional to the rotation rate. The value of MR is constant. The parameters of I_s are adjusted following the rotation rate in the simulation. The current parameter in simulation may be lower than the actual value, which may result in a slower initial increase in the simulated voltage compared to the experimental results.

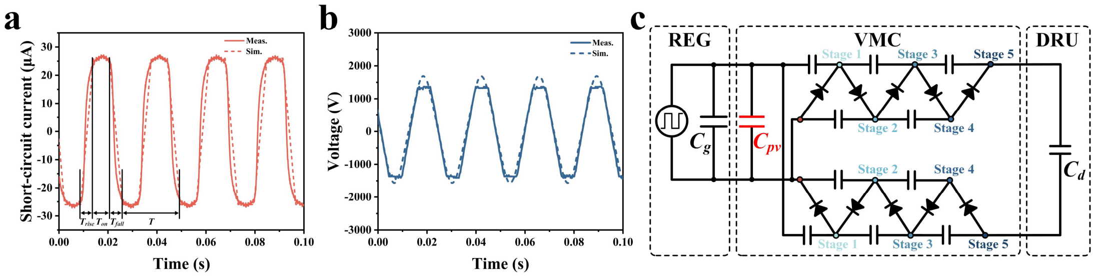

Fig. S28 (a) Simulated and Measured I_{sc} with MR = 45%. (b) Simulated and measured voltage of the REG after connecting the VMC. (c) Modified equivalent circuit of the ADRS.

Table S5 Parameters of the REG, VMC and C_d

Parameters of REG	
C_g (pF)*	31
r_0 (inner radius, mm)	17
r (outer radius, mm)	75
n (numbers of the electrode pairs)	4

g_0 (gap between the adjacent electrodes, mm)		2.0
I_s (600 rpm)	Amplitude (μA)	25.65
	T (ms)	23.6
	MR	45%
	T_{rise} and T_{fall} (ms)	0.5 × MR × Tperiod
	T_{on} (ms)	0.5 × (1-MR) × Tperiod
Parameters of VMC		
C_{pv} (pF)		40
C_0 (nF)		1
Parameters of C_d (DRU)		
C_d (pF)*		10

* The values used in the simulation for C_g and C_d are the actual ones measured by a precision LCR meter (TH 2816A, CN).

Note S5: Wind-driven dust removal experiments in the real environment

The REG was driven by the natural wind with a speed between 0 ~ 4.5 m/s. Movie S4 illustrates that two DRUs for two solar panels driven by one REG and VMC can almost remove Ulanbuhe-1 dust effectively after about 30 minutes. Movie S5 shows the comparison of the dust removal performance with and without the DRU. It was found that after about 20 minutes, the dust on the solar panel with the DRU was almost removed, while the dust still accumulated on the solar panel without the DRU. In the experiments, the power supply was used to power the homemade current testing board and the light intensity sensor.

Note S6: Experiment on the dust removal effect with different connection modes

In one test, the solar panel is initially left non-dust for a while after the installation of DRU. Subsequently, sand dust is uniformly spread onto the surface of the solar panel. After the dust spread is completed and a period to allow the solar panel output to stabilize, REG starts to work, and then dust is removed from the solar panel. Before the initiation of each experiment, the solar panel is thoroughly cleaned, and the initial conditions are kept consistent across the four experiments. The solar panels used in the

test under different connection types and stability under high voltages have a smaller size compared to panels in the demonstration experiment. They have an area of 27 cm × 20 cm, a peak power of 5 W, and a peak voltage of 18 V.

Note S7: Experiment on the influence of high voltage on solar panel output

Two solar panels of the same model are used, with the front electrode and back electrode connected to the output terminal of VMC, respectively (**Fig. S29**). One cycle of the test process is: after REG starts working for about 3 hours, disconnect the solar panel from VMC, and measure the current output of the solar panel. After the measurement, the charge in all the capacitors on VMC and C_d is released, and then the solar electrode is connected to VMC again. Repeat the measurements for multiple cycles per day to simulate the conditions in real application scenarios. Because the wind in the real environment is intermittent, the system may also run repeatedly.

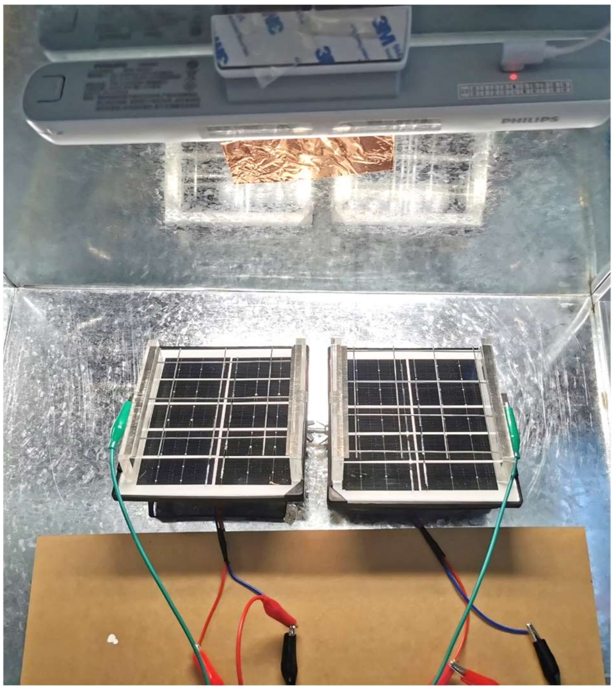

Fig. S29 Photograph of the experimental setup for influence test of the high voltage on the solar panel output.

References

S1. M. Bi, S. Wang, X. Wang, X. Ye, Freestanding-electret rotary generator at an average conversion efficiency of 56%: Theoretical and experimental studies. *Nano Energy* **41**, 434–442 (2017).

S2. Z. Cao, S. Wang, M. Bi, Z. Wu, X. Ye, Largely enhancing the output power and charging efficiency of electret generators using position-based auto-switch and passive power management module. *Nano Energy* **66**, 104202 (2019).

Supplementary Movie Description

Movie S1

Movie S1 shows the SiO_2 dust particles with an average diameter of 110 μm repelling from the glass cover with a commercial power supply of 4.5 kV. The DRU is placed horizontally without any inclination. The gap *g* between the mesh electrode and the lower electrode is 10 mm, and the mesh electrode pitch distance *L* is 10 mm.

Movie S2

Movie S2 shows the removal process of the SiO_2 dust particles with an average diameter of 110 μm repelling from the glass cover with the ADRS. The DRU is tilted with an angle of 10 degrees, *g* is 15 mm and *L* is 20 mm.

Movie S3

Movie S3 shows the wind-driven application demonstration of the ADRS prototype. The dust removal experiments are conducted for real solar panels under the window in a laboratory. Under the low wind speed of 1.6 m/s, two DRUs for two solar panels driven by one REG and VMC have been shown to remove most dust effectively without any external power supply within a short time of 6.6 minutes.

Movie S4

Movie S4 shows the wind-driven application demonstration of the ADRS prototype in the real environment. The dust removal experiments are conducted for real solar panels on the roof of a building at Tsinghua University. The REG was driven by the natural wind with a speed between 0 ~ 4.5 m/s. Two DRUs for two solar panels driven by one REG and VMC can almost remove Ulanbuhe-1 dust effectively after about 30 minutes.

Movie S5

Movie S5 shows the wind-driven application demonstration of the ADRS prototype in the real environment. It shows the comparison of the dust removal performance with

and without the DRU. The dust removal experiments are conducted for real solar panels on the roof of a building at Tsinghua University. The REG was driven by the natural wind with a speed between 0 ~ 4.5 m/s. After about 20 minutes, the dust on the solar panel with the DRU was almost removed, while the dust still accumulated on the solar panel without the DRU.

Movie S6

Movie S4 shows the removal process of the SiO_2 dust particles with an average diameter of 15 μm with the ADRS. The DRU is tilted with an angle of 30 degrees, *g* is 15 mm, *L* is 10 and 20 mm. The dust is repelled and deposited on the central region of the mesh.
